# The epidemiology of the viral hepatitis in Brazil: A scoping review

**DOI:** 10.1371/journal.pone.0353840

**Published:** 2026-07-17

**Authors:** Cesar Augusto Inoue, Thais Sena de Paula Domingues, Maria Cecilia Araripe Sucupira, Roberta Sitnik, Caroline Thomaz Panico, Denize Ornelas Pereira Salvador de Oliveira, Isis Aleixo Barone Esquiçati, Ketti Gleyzer de Oliveira, Nathalia Villa dos Santos, Peter James Robinson, Raquel Queiroz de Araujo, Roberta dos Santos Pereira, Sabrina David Pugliese, Elton Carlos de Almeida, Carla Francisca dos Santos Cruz, Ana Paula Maciel Gurski, Maiko Luis Tonini, Flávia Moreno Alves de Souza, Bruna Emanuelle Alvarenga Fanis, Mário Peribañez Gonzalez, João Renato Rebello Pinho

**Affiliations:** 1 Hospital Israelita Albert Einstein, São Paulo, Brazil; 2 Departamento de HIV/aids, Tuberculose, Hepatites Virais e IST, Ministério da Saúde, Brasília, Distrito Federal, Brazil; UFSJ: Universidade Federal de Sao Joao del-Rei, BRAZIL

## Abstract

**Background:**

Brazil has advanced toward eliminating viral hepatitis by 2030 through broad prevention, diagnosis, and treatment policies. Yet substantial gaps persist across the care continuum, especially in vulnerable populations and underserved regions. This scoping review synthesizes nationwide epidemiologic patterns and identifies knowledge gaps to inform policy.

**Methods:**

We conducted a PRISMA‑guided scoping review on seven databases (2013–2024) for Portuguese/Spanish/English observational studies or systematic reviews with post‑2010 data, extracting epidemiological patterns by viral hepatitis type and population group.

**Results:**

Across 200 included studies, the combined sample encompassed more than 14.5 million individuals. Hepatitis B was the most studied, followed by hepatitis C. Hepatitis A seroprevalence in general population ranged from 33.3–67.8%. In vulnerable populations, highest seroprevalence was among incarcerated populations (88.1%), followed by immigrants (87.4%) and transgender individuals (75.6%). Hepatitis B presented HBsAg prevalence ranging from 0–7.5% in the general population, with the highest rates in an endemic Amazon region. Hepatitis C seroprevalence varied from 0.04–2.2% in the general population, with higher rates among people who use drugs (36.9%). Hepatitis D was predominantly reported in the Amazon region, with prevalence ranging from 0–23.9%. For hepatitis E, prevalence in the general population ranged from 0.9–59.4%, with the highest value in the South region.

**Conclusions:**

Viral hepatitis in Brazil shows heterogeneous epidemiologic patterns across regions and populations, alongside unequal research output. The predominance of small, cross‑sectional studies and their limited integration with social determinants of health highlight the need for future research employing methodologies that address the gaps identified in this review. The resulting evidence map can guide policies aligned with national elimination goals.

## Introduction

Viral hepatitis represents a major public health issue, accounting for high rates of morbidity and mortality worldwide. It is estimated that these infections result in approximately 1.34 million deaths per year, with the majority attributed to hepatitis B virus (HBV) and hepatitis C virus (HCV) infections [1,2]. Chronic forms of these infections are associated with severe clinical outcomes, such as liver cirrhosis and hepatocellular carcinoma, with HBV and HCV responsible for more than 50% of all liver cancer cases [1].

Brazil is estimated to be one of the countries with the highest viral hepatitis global burden, although national prevalence data remain limited [1,3]. Despite a decline in incidence and mortality rates over the years [4], largely due to the strengthening of public health policies such as the expansion of hepatitis B vaccination coverage and the incorporation of direct-acting antivirals for hepatitis C into the Brazilian Unified Health System (SUS), significant challenges remain in improving diagnostic coverage and access to treatment [3]. In addition, regional inequalities directly impact the country’s epidemiological landscape, underscoring the importance of health social determinants in shaping effective strategies for viral hepatitis prevention and control.

These disparities are particularly relevant given Brazil’s continental dimensions and its strategic importance as the largest country in Latin America, both in population and territory. As such, the country plays a central role in achieving hepatitis elimination goals in the region. Its national efforts are aligned with the goals of the Sustainable Development Agenda and the World Health Organization (WHO) Global Health Sector Strategy on Viral Hepatitis [5,6]. Aligned with these commitments, the country has adopted measures to accelerate the elimination of viral hepatitis as a public health problem. Notable initiatives include the establishment of the Interministerial Committee for the Elimination of Tuberculosis and Other Socially Determined Diseases (Comitê Interministerial para a Eliminação da Tuberculose e de Outras Doenças Determinadas Socialmente – Cieds) [7] and the creation of the program “Brasil Saudável” [8], both aimed at promoting intersectoral actions to mitigate social vulnerabilities and reduce health inequities, particularly among historically neglected populations.

Considering this context, this study proposes a scoping review of viral hepatitis A, B, C, D, and E epidemiology in Brazil over the last decade. The objective is to map epidemiological patterns, identify regional disparities and vulnerable populations, and uncover critical knowledge gaps. By consolidating and critically analyzing scientific literature, this review aims to support evidence-based public health policies and to highlight areas requiring further investment in research and epidemiological surveillance.

## Methodology

### Development of research questions using the “PCC Strategy”

To conduct this scoping review, three guiding questions were formulated based on the central theme – “Epidemiology of viral hepatitis”:

1. What evidence/information currently exists on the epidemiological profile and regional distribution of viral hepatitis cases in Brazil?
2. What evidence/information currently exists on the global burden of viral hepatitis in Brazil?
3. What evidence/information currently exists on the epidemiological profile and regional distribution of viral hepatitis cases in vulnerable populations in Brazil?

These questions were structured following the PCC strategy (Population, Concept, and Context), as recommended by the Joanna Briggs Institute (JBI) for formulating scoping review questions [9].

### Standardization and search strategy

This scoping review was conducted according to JBI methodology [9,10] and reported following the PRISMA-ScR (Preferred Reporting Items for Systematic Reviews and Meta-Analyses Extension for Scoping Reviews) guidelines [11]. A protocol was developed for this scoping review, including the data extraction framework, prior to the conduct of the review. The protocol guided the study selection and data extraction processes. It was subsequently registered on the Open Science Framework platform (https://doi.org/10.17605/OSF.IO/NFUMV) during the review process and updated to reflect the final methodological approach.

To ensure comprehensive search, Medical Subject Headings (MeSH), and text words were used, applied to titles, abstracts, and keywords (Table 1). The strategies were adapted according to each database structure (S1 Table).

**Table 1:** Descriptors and free terms used in the scoping review strategy.

Themes	MeSH/Text words
Viral hepatitis	*Hepatitis, Viral, Human*
Hepatitis A	*Hepatitis A; Hepatitis A Vaccines; Hepatitis A virus; Hepatitis A Virus, Human; HAV*
Hepatitis B	*Hepatitis B; Hepatitis B virus; Hepatitis B Vaccines; Hepatitis B, Chronic; Acute Hepatitis B; HBV*
Hepatitis C	*Hepatitis C; Hepatitis C, Chronic; Acute Hepatitis C; HCV RNA; HCV*
Hepatitis D	*Hepatitis D; Hepatitis Delta Virus; Hepatitis D, Chronic; Delta Superinfection; HDV RNA; HDV*
HepatitisE	*Hepatitis E virus; Hepatitis E; HEV RNA; HEV*
Epidemiology	*Epidemiology; Prevalence; Incidence; Mortality; Morbidity; Seroepidemiologic Studies; Reinfection; Epidemiologic Studies; Vaccination Coverage; Transmission; viraemic rate; therapy rate; healing rate; vaccination rate; Lethality.*
Global burden of viral hepatitis	*Carcinoma, Hepatocellular; Liver Failure, Acute; Liver Cirrhosis; Liver Transplantation; Hepatic Insufficiency; Acute-On-Chronic Liver Failure; Infectious Disease Transmission, Vertical; Disability-adjusted life years; Global burden of disease*
Vulnerable populations	*Vulnerable population; Emigrants and Immigrants; Transients and Migrants; Refugees; Health Personnel; Homosexuality; Bisexuality; Transgender people; Transplant recipients; Pregnant Women; Indigenous Peoples; Substance Abuse Detection; Intravenous Substance Abuse; Drug users; Minority Health; Sex Offenses; Infectious Disease Transmission, Vertical Blood Component Transfusion; Liver Diseases; Transgender Persons; Sexual and Gender Minorities; Black or African American; Renal Dialysis; Adolescent; Black People; Sex workers; Alcohol Drinking; Addiction, Substance; Pre-Exposure Prophylaxis; Post-Exposure Prophylaxis; Accidents, Occupational; Condoms; Immunocompromised Host; Prisoners; Diabetes Mellitus; Hypertension; HIV Infections; Pregnancy Complications, Infectious; Mental Disorders; Family Planning Policy; Substance-Related Disorders; Ill-Housed Persons; Homeless; Amazon; Riparian population; Household contacts; Quilombola Communities; Beauty Centers; Aesthetics Centers; Gender-Nonconforming Persons*

The databases consulted were PubMed, Virtual Health Library (BVS), EMBASE, SciELO, Scopus, Web of Science, and CINAHL. The final search was conducted on 16th April 2024 and was restricted to references published between January 1, 2013, and March 15, 2024, containing data produced after January 1, 2010.

### Selection of articles and sources of evidence

The inclusion and exclusion criteria are described in Table 2.

**Table 2:** Inclusion and exclusion criteria.

Inclusion criteria:
Publications in Portuguese, Spanish, and English;
Full-text available studies;
Peer-reviewed studies, technical reports, theses, dissertations, and institutional documents;
Observational studies, including cross-sectional, cohort, case-control, case series, and systematic reviews;
Studies published between January 1, 2013, and March 15, 2024, containing data produced after January 1, 2010.
**Exclusion criteria:**
Studies published before 2013 or with data collected before 2010;
Protocols without data, single case reports, narrative reviews, and editorials;
Studies exclusively addressing virological, clinical aspects, vaccine trials, and transplants.

### Selection of texts included in the scoping review

Article selection followed a structured three-step process. Initially, references were organized in EndNote v.20 software [12], with duplicate removal. Then, two independent reviewers screened titles and abstracts in the Rayyan platform [13], excluding studies irrelevant to the research questions. Finally, selected texts underwent full-text reading to confirm eligibility. In cases of disagreement, a third reviewer was consulted to resolve conflicts [10].

### Data extraction from selected texts

Data extraction followed JBI guidelines, using a standardized form in the REDCap platform [14,15]. The form included variables such as publication type, study characteristics (author, year, location, methodological design), target population characterization (age group, sex at birth, race/ethnicity, and education level), sample size, epidemiological data (prevalence, incidence, mortality, vaccination coverage).

Populations included in this scoping review were categorized based on official documents from the Brazilian Ministry of Health [16] and grouped by similarity, considering epidemiological characteristics and associated vulnerabilities. The classification was structured into six categories with operational definitions as follows. (1) General population and proxy groups were defined as samples intended to approximate the broader community distribution or routinely screened groups used as proxies for population-level estimates (for example, blood donors and pregnant women). (2) Age groups were defined as samples explicitly restricted to a specific age range (for example, children or older adults). (3) Clinical populations were defined as groups characterized by clinical conditions or healthcare-related exposures that may modify viral hepatitis risk, testing pathways, or disease susceptibility; this category included individuals with chronic diseases, or liver diseases, those on hemodialysis, transplant recipients, people with a history of blood transfusion, people living with HIV/aids (PLWHA), and individuals with alcohol abuse. (4) Occupational risk groups were defined as workers whose professional activities entail potential exposure to blood or body fluids, sharp injuries, or contaminated materials, this category included healthcare workers, urban sanitation/waste collection workers, beauty sector workers, and public safety personnel. (5) Remote area populations were defined as communities living in geographically remote settings or with limited access to health services and surveillance, including rural, riverside, *quilombola*, and Indigenous communities. (6) Socially and structurally vulnerable populations were defined as groups experiencing social exclusion, marginalization, or structural barriers to prevention, diagnosis, and care, which may increase exposure or reduce access to timely services, this category included gay and men who have sex with men (MSM), transgender individuals, immigrants and refugees, incarcerated individuals, homeless people, people who use drugs (PWUD), and sex workers. Additionally, in line with national surveillance reports and clinical guidelines, we defined a seventh analytic stratum called Amazonian populations. This stratum covers the Legal Amazon region and includes urban, rural, riverine, and Indigenous communities which are prioritized for viral hepatitis prevention and screening [16–19].

Epidemiological data were collected and extracted following the established standardization. Discrepancies between reviewers were resolved through the same process as described for study selection. In cases of overlap between population groups, where the study did not present specific values for each population, prevalence was assigned to multiple groups, ensuring the representativeness of the different contexts analyzed.

### Data analysis

Extracted data were analyzed descriptively and categorized according to the variables defined in the extraction form. The primary analytical approach incorporated key variables including publication characteristics, study design methodology, geographic coverage of data collection (whether national or regional in scope), the hepatitis virus type being examined, and the target population under investigation. Each study was classified based on the population groups previously established by the reviewers. In cases of overlap, such as studies involving multiple groups simultaneously (e.g., transgender incarcerated individuals, sex workers residing in the Amazon region, gay men and MSM living with HIV/aids), all relevant categories were assigned, allowing for a more comprehensive analysis.

Data extraction was followed by an analysis plan development aggregating information on all viral hepatitis types and subsequently stratifying them by specific hepatitis type. Results were presented according to the number of references addressing each variable in the extraction form. Since a single study could report multiple variables, the total records per category did not necessarily correspond to the total number of studies included in the review.

Epidemiological data, such as prevalence, incidence, and mortality, were extracted from the articles based on findings consistent with viral hepatitis infection, confirmed by serological testing or medical records. The epidemiological data were systematized according to the specific serological markers for each type of viral hepatitis, with these values being used for analysis within each population group defined in the scoping review design. To optimize result presentation, a graph was developed for each hepatitis type containing study identification, publication year, and sample size.

As a scoping review, this study mapped descriptive epidemiological data and did not pursue meta-analyses, risk factor hierarchies, or causal inference, which fall under analytical epidemiology, although the extracted data included information on prevalence. Described results prioritized the most relevant prevalence data; therefore, not all studies in this review were presented in the text.

## Results

### PRISMA flowchart description

**Fig 1:**
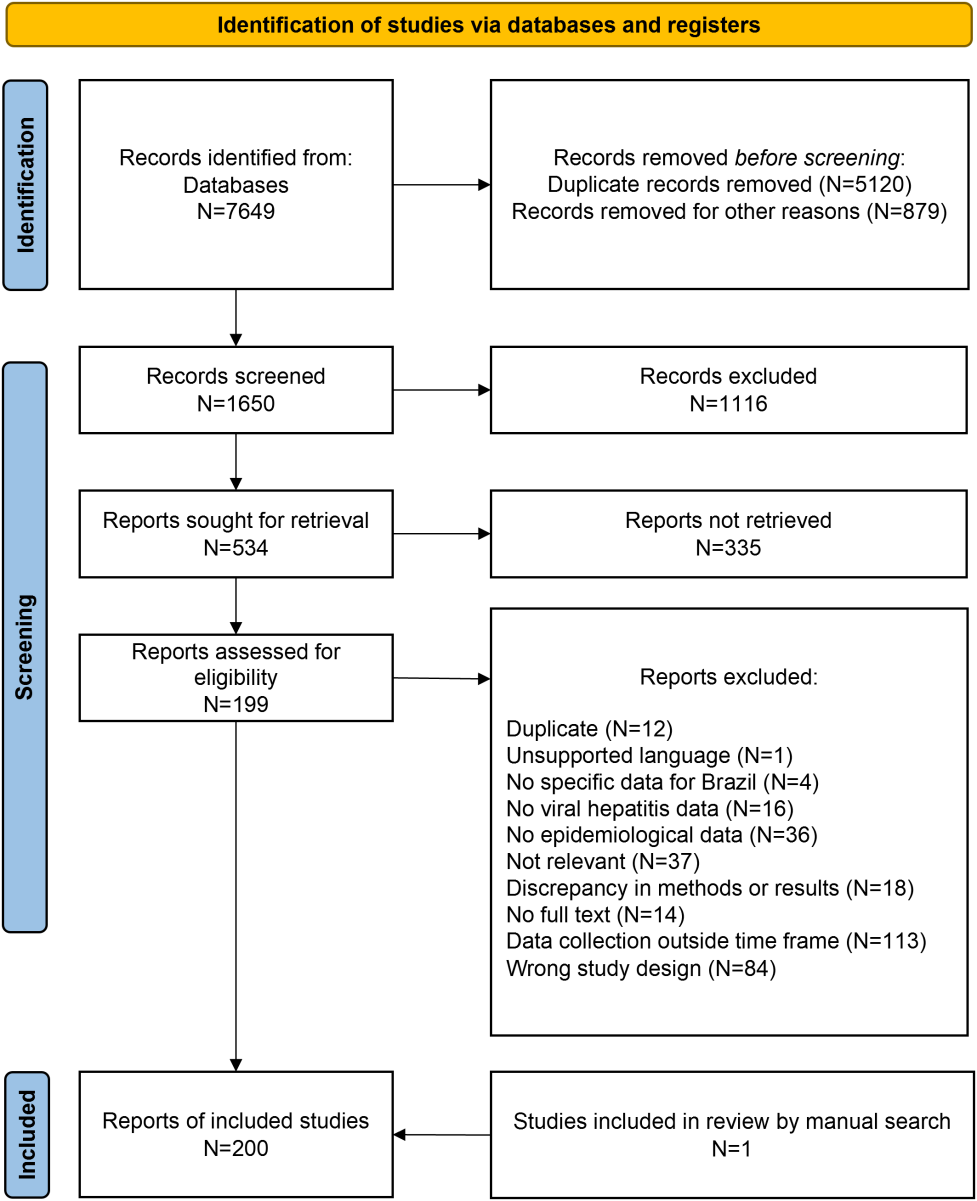
Flowchart of the study selection.

The search in the PubMed, LILACS, EMBASE, Cochrane Library, Scopus, Web of Science, CINAHL databases, and gray literature resulted in the identification of 7,649 references. After removing 5,999 records (5,120 duplicates, 790 data collection outside time frame, 88 without abstracts, and 1 in an unsupported language), 1,650 references were selected for title and abstract screening (Fig 1 and S1 Table).

At this stage, 1,116 articles were excluded according to the pre-established eligibility criteria. The remaining 534 articles underwent full-text reading, leading to the exclusion of 335 studies. Among the exclusion reasons, 12 articles were considered duplicates at this stage, mainly gray literature and conference abstracts whose full texts were identified in the final selection. One study was excluded for being in a language incompatible with the scope criteria. Four articles did not present data on viral hepatitis epidemiology in Brazil, while 16 studies did not address viral hepatitis. Thirty-six articles lacked relevant epidemiological information. Another 37 studies were discarded for not presenting pertinent data to the analysis, and 18 were excluded due to poor methodological quality or scientific rigor. Additionally, 14 texts were unavailable in full, 113 studies were excluded for having a data collection period outside the inclusion criteria, and 84 articles were not original publications or presented study design incompatible with the criteria of this review. In the end, 200 studies were included, one of which was identified through manual search (Fig 1).

### General information

**Fig 2:**
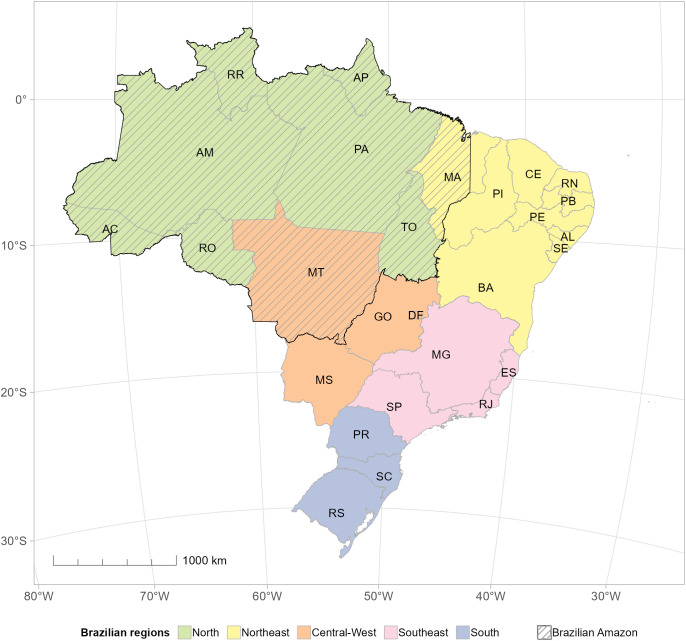
Brazil’s geopolitical map: macro-regions and states ^A, B^. (A) Map authored by the authors in R from IBGE vector boundary data. No basemap or satellite imagery was used. Data source: Brazilian Institute of Geography and Statistics (IBGE) (accessed on 19 Jan 2026): https://www.ibge.gov.br/en/geosciences/downloads-geosciences.html?lang=en-GB. (B) State abbreviations by Brazilian macro-region: North: AC – Acre; AM – Amazonas; AP – Amapá; PA – Pará; RO – Rondônia; RR – Roraima; TO – Tocantins. Northeast: AL – Alagoas; BA – Bahia; CE – Ceará; MA – Maranhão; PB – Paraíba; PE – Pernambuco; PI – Piauí; RN – Rio Grande do Norte; SE – Sergipe. Central-West: DF – Federal District; GO – Goiás; MS – Mato Grosso do Sul; MT – Mato Grosso. Southeast: ES – Espírito Santo; MG – Minas Gerais; RJ – Rio de Janeiro; SP – São Paulo. South: PR – Paraná; RS – Rio Grande do Sul; SC – Santa Catarina.

Among the 200 articles, the period between 2017 and 2020 (n = 92) had the highest number of included texts. Regarding publication type, 173 references were peer-reviewed original articles, followed by short communications (n = 15), conference abstracts (n = 9), and dissertations and theses (n = 3). Almost all included articles (n = 181) were cross-sectional studies, 6 were cohort studies, 4 were ecological studies, 3 employed mixed methodology (cross-sectional study combined with a cohort study), and 3 were based on secondary data/surveillance data. Few studies had national coverage (n = 13), while most had regional reach, distributed in descending order across the following regions: Southeast, Central-West, North, Northeast, and South (Fig 2).

Collectively, the evidence base comprises more than 14.5 million participants. Sample sizes varied widely overall (minimum 38; maximum 11,397,607; median 464). Hepatitis A showed the broadest range (51–11,397,607; median 523; 25 studies), while hepatitis B contributed to the largest evidence base (115 studies; 56–1,991,120; median 435). Hepatitis C had the highest median sample size (median 600; 81–1,991,120; 91 studies). In contrast, hepatitis D study sizes were consistently low, with no dataset exceeding 4,000 participants (38–3,983; median 560.5; 16 studies). Hepatitis E study sizes were also limited, all below 3,000 participants (150–3,000; median 434; 30 studies).

Most studies were conducted exclusively with adults (n = 122), while 57 studies included all age groups, and 11 focused on children and young people. Data on age group were available in 192 texts, while 10 studies indicated age as an inclusion criterion without presenting specific analyses on this variable. Nearly all studies provided data on sex at birth (n = 192), and 124 texts included information on education level. However, only 89 studies addressed race/skin color.

Regarding the viral hepatitis investigated, hepatitis B was the most studied (n = 115), followed by hepatitis C (n = 91), hepatitis E (n = 30), hepatitis A (n = 25) and hepatitis D (n = 16). Some studies addressed more than one viral hepatitis, so the total number of texts presented in this result exceeds the total number of selected texts.

It is important to note that epidemiological estimates are based on different serological markers depending on hepatitis type, reflecting distinct aspects of infection (e.g., current infection or past exposure), and should therefore be interpreted within the context of each marker.

### Hepatitis A

Twenty-five texts included in this review addressed hepatitis A, with studies conducted between 2014 and 2023. Among them, four had national coverage, while the others (n = 21) were regional, predominantly in the Southeast region, followed by the Central-West, North, and South region.

**Fig 3:**
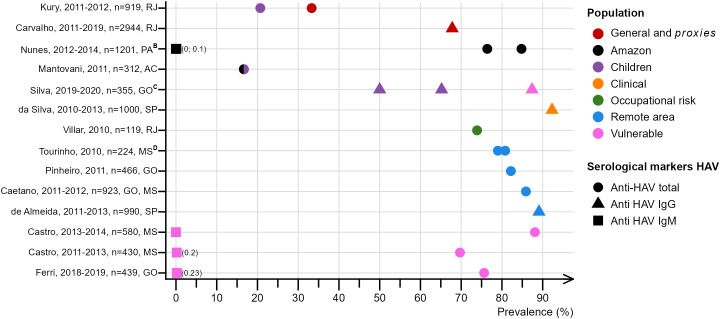
Evidence map of hepatitis A prevalence estimates in Brazil by population group and marker, 2013–2024^A^. (A) Each point is one extracted prevalence estimate, grouped by population category and ordered by year of data collection. Labels indicate first author, data-collection period, sample size, and study location (Brazilian state/UF). Study locations (states/UF) correspond to those shown in Fig 2. When a study reports more than one eligible estimate (e.g., for different population subgroups, settings, or assays), each estimate is plotted as a separate point and indicated with suffixes (e.g., “a/b”). When stratum-specific estimates are unavailable for overlapping populations, the same estimate may be displayed in more than one category. See Methods and S2 Table (supplementary dataset) for point-level details. (B) Canaã city, Pará State (Anti-HAV IgG: 76.4%; Anti-HAV IgM: 0.1%); Curionópolis city, Pará State (Anti-HAV IgG: 84.8%; Anti-HAV IgM: 0%). (C) 10–19 years (50%); ≤ 9 years (65.2%). (D) Serum sample (79.01%); Oral fluid (80.8%).

The general population was analyzed in two studies, with anti-HAV IgG/anti-HAV total seroprevalence ranging from 33.3% to 67.8% [20,21], both conducted in the Southeast region (Rio de Janeiro). The lowest value was identified in a young population aged 15–19 years [20] (Figs 2 and 3).

Among children, anti-HAV total seroprevalence ranged from 16.6% to 20.7% [20,22]. In a study involving Latin American immigrants and refugees, anti-HAV IgG prevalence among children was 50% − 65.2% [23] (Fig 3).

Analysis of specific populations revealed higher seroprevalence values. Among individuals living in remote areas, total anti-HAV ranged from 79.01% to 85.9% [24–26], while anti-HAV IgG reached 89.1% [27], with all studies conducted regionally in the Central-West and Southeast regions (Figs 2 and 3).

Among individuals with specific clinical conditions, a study conducted in a tertiary care service identified an anti-HAV IgG seroprevalence of 92.3% in people with chronic hepatitis C [28]. In the occupational risk population, anti-HAV total was 73.9% [29] (Fig 3).

In populations with social/structural vulnerabilities, anti-HAV total seroprevalence was 75.63% in transgender women/*travestis* [30] and 69.7% in gay men and MSM [31]. Additionally, in these groups, anti-HAV IgM, a marker of recent infection, was identified in 0.23% of trans people [30] and 0.2% of gay men and MSM [31]. In a study with Latin American immigrants and refugees, anti-HAV IgG seroprevalence was 87.4%, with the highest values observed among Haitians (94.9%) [23]. Anti-HAV total seroprevalence was 88.1% in the incarcerated population [32] (Fig 3).

In the Amazonian population, anti-HAV total values varied widely, ranging from 16.6% to 84.8% [22,33]. The highest prevalence was identified in the state of Pará, in areas with intense migratory flows due to mining exploitation, while the lowest value was observed in a study focused on children in the Amazon region (Figs 2 and 3).

### Hepatitis B

Hepatitis B was addressed in 115 publications, with studies conducted between 2013 and 2024. Four studies presented national data, while the others had regional coverage, with most of them conducted in the Southeast region, followed by the Central-West, Northeast, North, and South regions (Fig 2).

**Fig 4:**
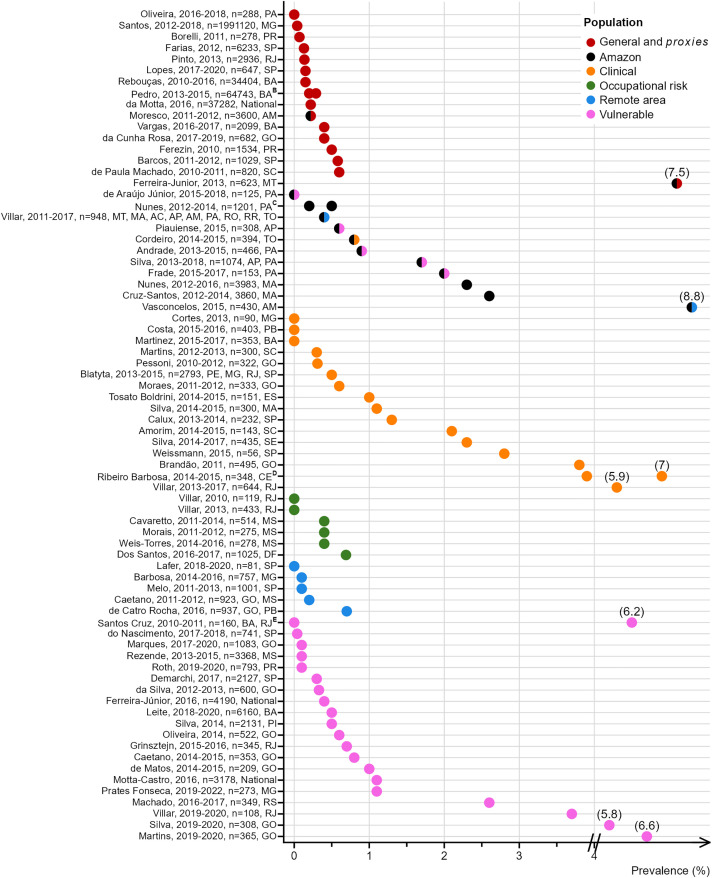
Evidence map of hepatitis B (HBsAg) prevalence estimates in Brazil by population group, 2013–2024^A^. A) Each point is one extracted prevalence estimate, grouped by population category and ordered by year of data collection. Labels indicate first author, data-collection period, sample size, and study location (Brazilian state/UF). Study locations (states/UF) correspond to those shown in Fig 2. When a study reports more than one eligible estimate (e.g., for different population subgroups, settings, or assays), each estimate is plotted as a separate point and indicated with suffixes (e.g., “a/b”). When stratum-specific estimates are unavailable for overlapping populations, the same estimate may be displayed in more than one category. See Methods and S2 Table (supplementary dataset) for point-level details. (B) Southeastern region of Bahia State (0.2%); Southern region of Bahia State (0.29%). (C) Canaã city, Pará State (0.2%); Curionópolis city, Pará State (0.5%). (D) Clinical population: PLWHA (3.9%); Hemodialysis (7%). (E) Rio de Janeiro city, Rio de Janeiro State (6,2%); Salvador city, Bahia State (0%).

**Fig 5:**
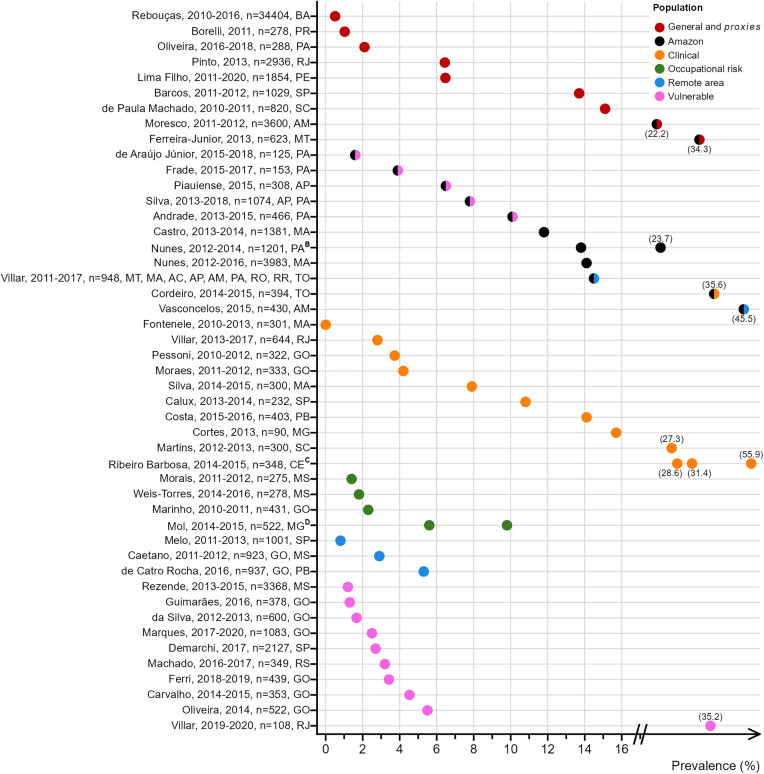
Evidence map of hepatitis B (anti-HBc total) prevalence estimates in Brazil by population group, 2013–2024^A^. (A) Each point is one extracted prevalence estimate, grouped by population category and ordered by year of data collection. Labels indicate first author, data-collection period, sample size, and study location (Brazilian state/UF). Study locations (states/UF) correspond to those shown in Fig 2. When a study reports more than one eligible estimate (e.g., for different population subgroups, settings, or assays), each estimate is plotted as a separate point and indicated with suffixes (e.g., “a/b”). When stratum-specific estimates are unavailable for overlapping populations, the same estimate may be displayed in more than one category. See Methods and S2 Table (supplementary dataset) for point-level details. (B) Canaã city, Pará State (13.8%); Curionópolis city, Pará State (23.7%). (C) Clinical population: PLWHA (28.6%); Coagulopathy patients (31.4%); Hemodialysis (55.9%). (D) Occupational risk population: Domestic waste workers (5.6%); Healthcare waste workers (9.8%).

In the general population and proxy groups (youth, pregnant women, and individuals over 50 years old), HBsAg prevalence was reported in 16 regional studies [34–49] and one national study [50]. The values ranged from 0% to 7.5%, with the highest prevalence observed in a population from the Western Amazon, an area considered endemic [41]. The national study, conducted with young members of the Armed Forces, found a prevalence of 0.22% [50] (Figs 2 and 4). Meanwhile, anti-HBc seroprevalence in the general population varied widely, from 0.51% to 34.3% [34–38,41,43,45,46,51] with the highest value also recorded in the Western Amazon [41]. Among individuals over 60 years old, anti-HBc prevalence ranged from 13.7% to 15.1% [35,38] (Figs 2 and 5).

In clinical populations, HBsAg prevalence was reported in 19 studies [52–70]. Notably: patients undergoing hemodialysis (0.8%−7%), blood transfusion recipients (0%−0.5%), individuals with mental disorders (0.65%−1.1%), and PLWHA, had coinfection HBV (HBsAg)/HIV rates from 0.3% to 3.9% [52–70]. Anti-HBc seroprevalence was identified in 11 studies, with the widest range observed in hemodialysis patients (0%−55.9%) [56,60,65,69]. In a study with a convenience sample from a dialysis service in the Northeast, prevalence reached 55.9% [65], while in the North, it was 35.6% [56] (Figs 2 and 4). In blood transfusion recipients, anti-HBc prevalence was 31.4%, with most participants having received blood before 1994. HBV (anti-HBc)-HIV coinfection ranged from 10.8% to 28.6% in PLWHA. Among individuals with alcohol abuse, prevalence ranged from 1.6% to 15.7% [57,59], while in individuals with mental disorders, it varied from 4.2% to 7.9% [63,66] (Fig 5).

Nine studies evaluated workers exposed to occupational risk for hepatitis B. Healthcare professionals had 0.4% and 1.4% HBsAg and total anti-HBc prevalence, respectively [71]. Urban cleaning and hospital waste workers had the highest HBsAg prevalence (0.4% to 0.7%) [72,73], while anti-HBc total ranged from 1.8% to 9.8% [73–75]. Beauty and aesthetics workers were mentioned in two studies, with HBsAg ranging from 0% to 0.4% [29,76]. For public security professionals, values ranged from 0% to 0.22% [50,77] (Figs 4 and 5).

In remote area populations, four studies in rural settings reported HBsAg prevalence ranging from 0.1% to 0.7% [24,78–80], while anti-HBc values varied from 0.8% to 5.3% [24,79,80], with the highest values for both markers identified among sugarcane cutters in the Northeast and Central-West [79]. Riverside populations had HBsAg prevalence between 0.9% and 2% and anti-HBc total between 3.9% and 10.1% [81,82], with overlapping vulnerabilities in both groups, including sex workers [82] and PWUD [81]. Indigenous populations were analyzed in three studies, with HBsAg ranging from 0% to 8.8% [83–85] and anti-HBc between 14.5% and 45.5% [84,85], with the highest endemicity among Yanomami Indigenous people [84] (Figs 2, 4 and 5).

Twenty-eight studies addressed socially/structurally vulnerable populations. Among incarcerated individuals, HBsAg prevalence ranged from 0.04% to 2.6%, while anti-HBc ranged from 1.2% to 3.2% [86–92]. Among PWUD, values ranged from 0% to 6.2% for HBsAg and from 1.6% to 35.2% for anti-HBc [59,81,93–97]. Among immigrants and refugees, HBsAg prevalence ranged from 0.3% to 6.6% [98–100], with the highest value recorded in the Central-West [99]; for anti-HBc, it was 2.7% [98]. Among homeless individuals, prevalence ranged from 0.8% to 3.7% for HBsAg [97,101,102] and from 4.5% to 35.2% for anti-HBc [97,103], while in low‑income individuals the prevalence of anti‑HBc was 1.3% [104]. Among gay men/MSM, values ranged from 0.6% to 1.1% [105,106] for HBsAg and 5.5% for anti-HBc [106]; however, in a study with individuals on pre-exposure prophylaxis for HIV, the prevalence was lower (0.1%) [107]. Among transgender women/*travestis*, prevalence was 0.7% for HBsAg [108] and 3.4% for anti-HBc [30]. Among sex workers, values ranged from 0.4% to 2% for HBsAg [82,109] and 3.9% for anti-HBc [82]. (Figs 2, 4 and 5).

In Amazonian populations, hepatitis B markers display marked heterogeneity: HBsAg prevalence ranges from 0% to 8.8% across studies [33,41,43,56,59,81,82,84,85,94,96,110,111], with higher values among PWUD [81,94,96] and in hard to reach endemic settings such as Indigenous communities [84,85] and remote or rural areas with precarious socioeconomic conditions [41,110,111]. Anti-HBc prevalence is likewise heterogeneous, ranging from 1.6% to 45.5% [33,37,41,43,56,59,81,82,84,85,94,96,111], with particularly elevated values among Indigenous groups living in remote areas [84,85], residents of socioeconomically deprived remote settings [41,84], and clinical populations (e.g., patients on hemodialysis [56]). For both markers, the broad variability reflects overlapping vulnerabilities found in Amazonian studies, (Figs 4 and 5).

### Hepatitis C

Ninety-one publications addressed hepatitis C, with studies conducted between 2013 and 2024. Four articles had national coverage, while 87 were conducted at the regional level. The Southeast region had the highest number of studies, followed by the Central-West/North and Northeast/South regions.

**Fig 6:**
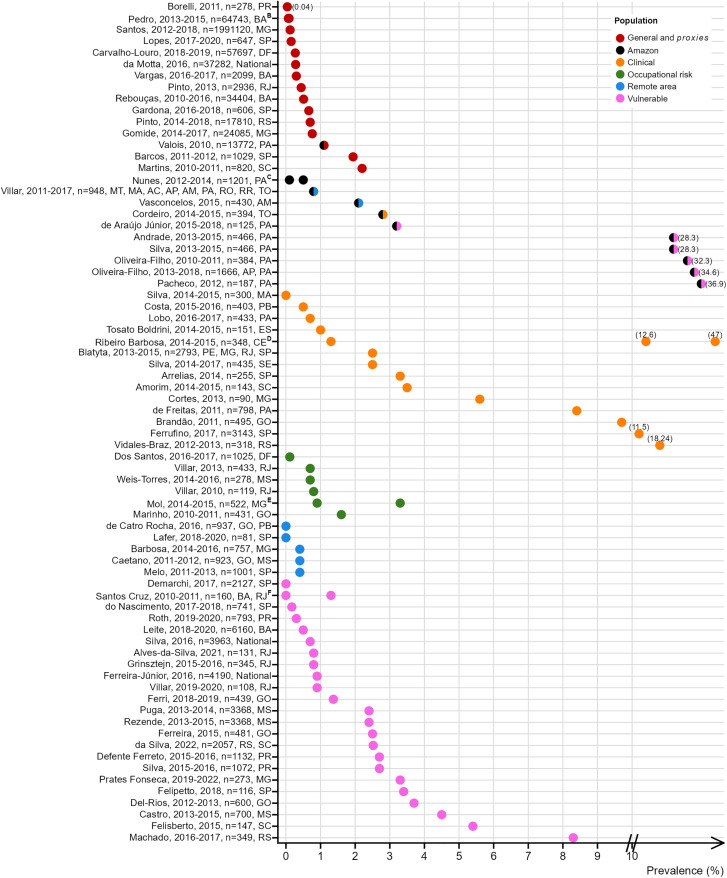
Evidence map of hepatitis C (anti-HCV) prevalence estimates in Brazil by population group, 2013–2024^A^. (A) Each point is one extracted prevalence estimate, grouped by population category and ordered by year of data collection. Labels indicate first author, data-collection period, sample size, and study location (Brazilian state/UF). Study locations (states/UF) correspond to those shown in Fig 2. When a study reports more than one eligible estimate (e.g., for different population subgroups, settings, or assays), each estimate is plotted as a separate point and indicated with suffixes (e.g., “a/b”). When stratum-specific estimates are unavailable for overlapping populations, the same estimate may be displayed in more than one category. See Methods and S2 Table (supplementary dataset) for point-level details. (B) Southeastern region of Bahia State (0.07%); Southern region of Bahia State (0.09%). (C) Canaã city, Pará State (0.1%); Curionópolis city, Pará State (0.5%). (D) Clinical population: PLWHA (1.3%); Hemodialysis (12.6%); Coagulopathy patients (47%). (E) Occupational risk population: domestic waste workers (0.9%); healthcare waste workers (3.3%). (F) 0% (Rio de Janeiro city – Rio de Janeiro State); 1.3% (Salvador city – Bahia State).

Seroprevalence of anti-HCV in general population and proxy groups (blood donors, elderly individuals >60 years old, pregnant women, and young adults) was identified in 14 studies, one with national and 13 with regional coverage [35,36,42,44–48,50,112–116]. Values ranged from 0.04% to 2.2%, with the highest prevalence observed within elderly individuals (>60 years old) in Santa Catarina state (South region) [115]. Among pregnant women, anti-HCV ranged from 0.07% to 0.7% [44,48,116], with the highest prevalence found in a study conducted in a South region hospital (Rio Grande do Sul state). The only national study was conducted among young members of the armed forces, identifying an anti-HCV prevalence of 0.28% [50] (Figs 2 and 6).

Clinical populations were addressed in 17 studies [52–54,56–59,65–68,117–121]. Anti-HCV seroprevalence among people with blood transfusion history or blood products use was investigated in two studies, with values ranging from 2.5% to 47% [53,65]. The highest prevalence was reported in a study conducted in a specialized service in the Northeast region, with a sample composed of patients diagnosed with coagulopathy, a history of sexually transmitted infections, and transfusions performed before 1994. Among individuals on hemodialysis, seroprevalence ranged from 2.8% to 18.24%, with the lowest value found in the North region (Tocantins state) and the highest in the South region (Rio Grande do Sul state). One study reported a 5.3% HCV RNA prevalence [118]. Seven studies assessed anti-HCV prevalence among PLWHA [52,54,65,67,68,119,120], with values ranging from 0.7% to 11.5%. The highest HIV-HCV coinfection rate was recorded in a study conducted in an outpatient clinic, predominantly involving men with possible sexual transmission [119]. Among people with alcohol abuse, seroprevalence ranged from 3.2% to 5.6% [57,59] (Figs 2 and 6).

Among populations at occupational risk, anti-HCV prevalence ranged from 0.11% to 3.3% [29,50,72,73,75,77,122], with waste collectors handling hospital waste presenting the highest prevalence (Fig 6).

For populations in remote areas including Amazonian populations, anti-HCV seroprevalence ranged from 0% to 28.3%. The lowest prevalences were identified in the rural population (0% to 0.4%) [24,78–80]. In Amazonian populations, anti-HCV prevalence showed wide heterogeneity, ranging from 0.1% to 36.9% across studies [33,56,59,81,84,85,123–127]. Most investigations assessed PWUD, among whom the highest prevalences were observed [26,123–126,128], Indigenous populations presented low anti-HCV prevalence (2.1%) [84], in opposition to the elevated HBV burden reported for these groups elsewhere in our analysis. The lowest anti-HCV prevalence was documented in a study conducted in mining/garimpo areas characterized by precarious socioeconomic conditions [33] (Figs 2 and 6).

Twenty-nine studies addressed populations in social and structural vulnerability situations. Incarcerated individuals were the most frequently analyzed, with anti-HCV prevalences ranging from 0.17% to 8.3% [86–88,90,91,129–134]. Among homeless people, values ranged from 0.9% to 3.4% [97,135,136]. PWUD focused studies reported prevalence rates ranging from 0% to 36.9% [59,81,95,97,123–126,137,138], predominantly from the North region [59,81,123–125]. A national study on female sex workers found a prevalence of 0.9% [109]. Seroprevalence among transgender people/*travestis* was investigated in three studies, with values ranging from 0.8% to 1.37%. A national study reported a prevalence of 0.7% among gay men/MSM [139], while a regional study with PrEP users, mostly MSM, found a prevalence of 0.3% [107] (Fig 6).

### Hepatitis D

Hepatitis D was the least studied, with 16 publications conducted between 2017 and 2024, primarily concentrated in the period from 2018 to 2022. Three studies had national coverage (two with an exclusive focus on genotypes and one with prevalence data), while 13 were conducted at the regional level. Most publications focused on the Legal Amazon regions, except for one study conducted with a clinical population in the Southeast region.

**Fig 7:**
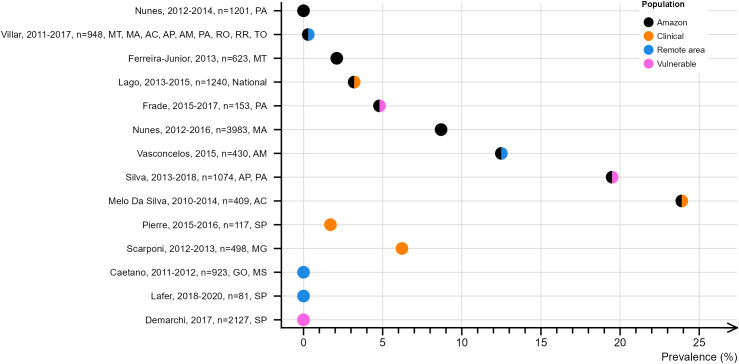
Evidence map of hepatitis D (anti-HDV) prevalence estimates in Brazil by population group, 2013–2024^A^. (A) Each point is one extracted prevalence estimate, grouped by population category and ordered by year of data collection. Labels indicate first author, data-collection period, sample size, and study location (Brazilian state/UF). Study locations (states/UF) correspond to those shown in Fig 2. When a study reports more than one eligible estimate (e.g., for different population subgroups, settings, or assays), each estimate is plotted as a separate point and indicated with suffixes (e.g., “a/b”). When stratum-specific estimates are unavailable for overlapping populations, the same estimate may be displayed in more than one category. See Methods and S2 Table (supplementary dataset) for point-level details.

Hepatitis D prevalence was assessed in individuals infected with the hepatitis B virus using the serological markers anti-HDV total or anti-HDV IgG. The highest prevalence variation was observed in the Amazonian/Legal Amazon population, ranging from 0% to 23.9%, making it the region with the largest number of studies [33,41,82,84,85,96,111,128,140]. The highest prevalence was recorded in a study conducted with patients with chronic hepatitis B treated at a specialized service in Acre state [128]. A high prevalence (19.5%) was also identified in a study among PWUD using the respondent-driven sampling (RDS) methodology [96]. In remote area populations, two studies on Indigenous populations reported prevalences between 0.3% and 12.5% [84,85]. Another study conducted in riverine communities in the Amazon region identified a prevalence of 4.8% among female sex workers [82] (Figs 2 and 7).

In the Southeast region, HDV prevalence was found to be 1.7% in a study of patients with chronic kidney disease undergoing hemodialysis or kidney transplantation [141] and 6.22% in a convenience sample for viral hepatitis testing in a reference public health laboratory [142]. A nationwide study estimated anti-HDV total prevalence by region [140], with the highest rate in the North region (8.5%), followed by Central-West (2.5%), Southeast (1.7%), and Northeast (0.8%). No HDV cases were identified in the South region (Figs 2 and 7).

Finally, three other studies conducted with Indigenous populations, Japanese immigrants, and rural populations did not detect serological markers for HDV [24,83,98] (Fig 7).

### Hepatitis E

Thirty texts addressed hepatitis E, with studies conducted between 2013 and 2024. All studies had regional coverage, with the highest number of publications in the Southeast region, followed by the Central-West, Northeast/South, and North regions.

**Fig 8:**
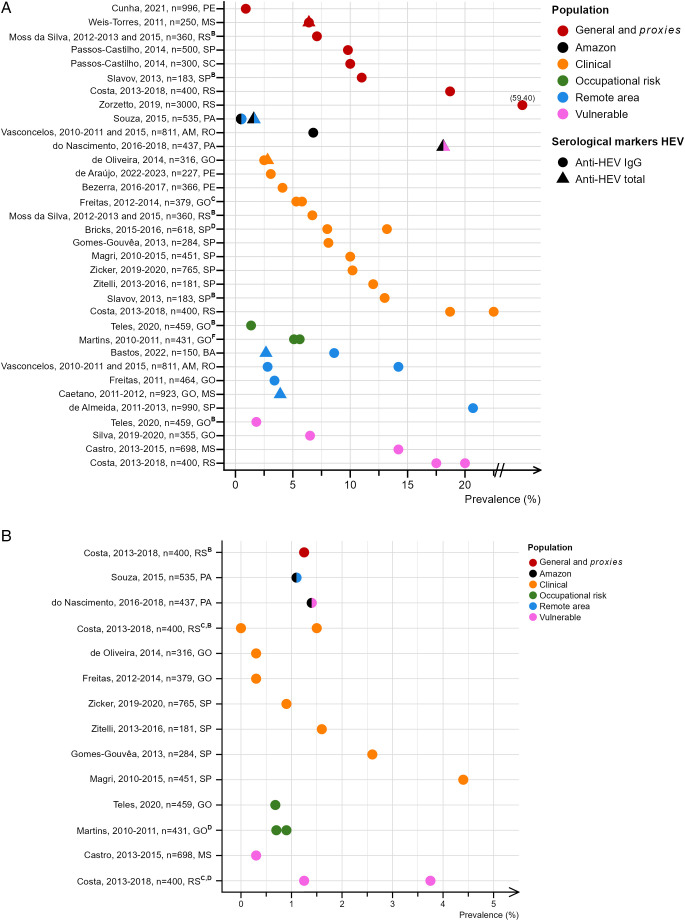
A. Evidence map of hepatitis E (anti-HEV IgG/total) prevalence estimates in Brazil by population group, 2013–2024^A,B^. (A) Each point is one extracted prevalence estimate, grouped by population category and ordered by year of data collection. Labels indicate first author, data-collection period, sample size, and study location (Brazilian state/UF). Study locations (states/UF) correspond to those shown in Fig 2. When a study reports more than one eligible estimate (e.g., for different population subgroups, settings, or assays), each estimate is plotted as a separate point and indicated with suffixes (e.g., “a/b”). When stratum-specific estimates are unavailable for overlapping populations, the same estimate may be displayed in more than one category. See Methods and S2 Table (supplementary dataset) for point-level details. (B) Repeated study labels indicate distinct estimates from the same study for different populations. (C) Immunoblot (5.3%); ELISA (5.8%). (D) Clinical population: Chronic hepatitis C without cirrhosis (8%); Chronic hepatitis C with cirrhosis (13.2%). (E) Clinical population: Transplant recipients (18.7%); Cirrhotic (22.5%). Vulnerable population: Residents of a low-income area (17.5%); PWUD (20%). (F) Immunoblot (5.1%); ELISA (5.6%). (G) Remote population: Indigenous (2.8%); Rural population (14.2%). **B. Evidence map of hepatitis E (anti-HEV IgM) prevalence estimates in Brazil by population group, 2013–2024**^**A,B**^. (A) Each point is one extracted prevalence estimate, grouped by population category and ordered by year of data collection. Labels indicate first author, data-collection period, sample size, and study location (Brazilian state/UF). Study locations (states/UF) correspond to those shown in Fig 2. When a study reports more than one eligible estimate (e.g., for different population subgroups, settings, or assays), each estimate is plotted as a separate point and indicated with suffixes (e.g., “a/b”). When stratum-specific estimates are unavailable for overlapping populations, the same estimate may be displayed in more than one category. See Methods and S2 Table (supplementary dataset) for point-level details. (B) Repeated study labels indicate distinct estimates from the same study for different populations. (C) Clinical population: Transplant recipients (0%); Cirrhotic (1.5%). Vulnerable population: low-income area residents (1.25%); PWUD (3.75%;). (D) Immunoblot (0.7%); ELISA (0.9%).

In the general population, eight studies identified anti-HEV IgG/total seroprevalence, ranging from 0.9% to 59.4% [143–150]. The highest value (59.4%) was observed in a study involving 3,000 participants from three major cities in Rio Grande do Sul state. According to the authors, this finding suggests possible endemicity in the region, possibly associated with a high density of swines [150]. Other studies were conducted among blood donors in the South, Southeast, Central-West, and Northeast regions, with variations between 0.9% and 18.7% [143–149] (Figs 2 and 8A).

In remote area populations, prevalence ranged from 0.5% to 20.7% [24,27,151–154]. The highest value was found in a rural community in São Paulo state, where the authors suggested that zoonotic exposure may have influenced the high seroprevalence, although without conclusive epidemiological evidence [27]. In other studies, prevalence ranged from 0.5% to 8.6% [24,151,152,154]. One study evaluated a *quilombola* community, reporting a 0.5% anti-HEV IgG prevalence [153]. Among Indigenous populations, the rate was 2.8% [154] (Figs 2 and 8A).

Anti-HEV IgG/total seroprevalence was also investigated in some clinical populations. Among individuals with transplantation history, prevalence ranged from 2.5% to 18.7% [143,155–157], with the highest prevalence study noting limitations due to a small sample size [143]. Among individuals with viral and non-viral origin chronic hepatitis, values ranged from 3.1% to 22.5% [143,158–162]. Additionally, one study reported a prevalence of 13% in individuals who had received blood transfusions. Among PLWHA, seroprevalence ranged from 4.1% to 6.7% [145,163] (Fig 8A).

Among socially and structurally vulnerable populations, prevalence values ranged from 14.2% to 20% for PWUD [143,164,165], with studies conducted in the South, North, and Central-West regions. It was 6.5% for Latin American immigrants and refugees in the Central-West region [23]; and finally, a prevalence of 1.82% was observed among people experiencing homelessness [166] (Figs 2 and 8A).

The prevalence of the acute infection marker for hepatitis E (anti-HEV IgM) was reported in 12 studies [143,153,155–157,160–162,164–167]. In the general population, a value of 1.25% was found [143]. In a remote area community, prevalence was 1.1% [153]. Among clinical populations, values varied according to the studied group, 0% to 2.6% in transplant recipients [143,155–157], and 0.3% to 4.4% in people with chronic hepatitis [160–162]. Among urban sanitation workers, prevalence ranged from 0.7% to 0.9% [166,167]. Finally, in vulnerable populations, a study assessed low-income individuals, reporting a seroprevalence of 1.25% [143], and in PWUD 0.3% to 3.8% [143,164,165] (Fig 8B).

## Discussion

The findings of this review reveal a heterogeneous viral hepatitis panorama in Brazil, highlighting regional inequalities, methodological gaps, and limitations in epidemiology incorporating health social determinants. The predominance of regional studies and the scarce national coverage suggest an uneven distribution of research output, possibly related to disparities in research funding and healthcare service infrastructure.

The geographical distribution of scientific evidence on viral hepatitis in Brazil showed discrepancies compared to the disease burden reported in the historical series of epidemiological bulletins [18]. While hepatitis A was more frequently studied in the Southeast and Midwest regions, data from the Brazilian Ministry of Health indicate a higher proportion of cases in the Northeast and North, suggesting an underrepresentation of these regions in research [18]. A similar situation was observed for hepatitis B, where the South region accounted for 31.2% of diagnosed cases between 2000 and 2023 but had one of the lowest numbers of studies [18]. Hepatitis C followed this trend, with the highest research output in the Southeast, while the South, which had the second-highest number of cases, had few studies [18]. For hepatitis D, most studies were concentrated in the North region, where most diagnosed cases are found. However, research on this infection remains scarce [18]. Hepatitis E showed a more aligned distribution between the number of cases and research efforts but remains under investigation, with heterogeneous prevalence estimates across studies [18]. These discrepancies underscore the need to expand epidemiology research not only in regions with a high disease burden, particularly in the North and Northeast, but also among vulnerable populations to ensure a more equitable understanding of viral hepatitis impact in Brazil.

The significant increase in publications between 2017 and 2020 may be related to intensified testing and treatment policies and epidemiological surveillance expansion during this period. Increased availability of rapid tests and direct-acting antiviral treatments for hepatitis C within the SUS, along with Brazil’s adherence to the WHO’s strategic plan to eliminate viral hepatitis as a public health problem by 2030, may have encouraged greater academic production on these topics. However, a decline in publication volume was observed in subsequent years, coinciding with the COVID-19 pandemic. This phenomenon reflects impacts on research output: disproportionate growth in COVID-19-related publications at the expense of non-COVID research, reallocation of research funding priorities, and pandemic-related disruptions to healthcare services [168–174]. In this context, viral hepatitis research publications may possibly have been affected by these intersecting factors. The economic contraction and the redirection of resources to other health emergencies may have compromised support for research, including viral hepatitis studies [174].

The heterogeneity of outcomes, study populations, research designs, methodologies employed, study locations, and sample sizes limited the comparability of the evidence, including across the population groups addressed and geographic regions. Methodologically, the predominance of small-scale cross-sectional studies represents a significant limitation in understanding the epidemiological patterns of viral hepatitis in Brazil. While cross-sectional studies are essential for estimating infection prevalence, their inability to establish causal relationships limits the exploration of risk factors and transmission dynamics. Moreover, small sample sizes compromise findings generalizability, particularly in the socioeconomic and geographical heterogeneity context.

Additionally, many studies relied on convenience samples, often recruited from specialized healthcare services, potentially overestimating infection prevalence due to selection bias, making extrapolation to the general population difficult. On the other hand, for hard-to-reach populations, such as people who use drugs, sex workers, and transgender individuals, methodologies like snowball sampling and respondent-driven sampling (RDS) have been widely used to overcome logistical and recruitment challenges [175,176]. However, these approaches also present limitations, such as dependence on the social networks of initial participants and difficulty ensuring population representativeness. The scarcity of population-based studies using probabilistic sampling reinforces the need for more methodologically robust investigations to enable a more precise characterization of viral hepatitis epidemiological determinants in Brazil.

The analysis of studied viruses revealed a concentration of research on hepatitis B and C, which may be explained by the disease burden associated with these infections and the greater availability of diagnostic tests [177]. In contrast, hepatitis D remained under-researched, with only 16 studies identified, reflecting both the underreporting of the infection and the limited laboratory infrastructure for its diagnosis [17,19,140,178]. Hepatitis A, in turn, showed wide variability in seroprevalence, highlighting changes in infection patterns in Brazil due to improved sanitation conditions and vaccine introduction [179,180]. This has led to reduced exposure in urban populations and a concentration of infections mainly in vulnerable groups, like incarcerated individuals, rural residents, and on specific outbreak situations, such as among gay and MSM populations [32,181].

Our synthesis reinforces the persistence of hepatitis B as a public health concern in Amazonian settings [182]. Heterogeneous HBsAg prevalence is observed. Studies describe overlapping vulnerabilities and potential transmission contexts, including cultural practices such as scarification, bloodletting, piercing and tattooing, early sexual debut, possible perinatal or early life exposures, and reduced access to vaccination in remote areas [84,85]. Perinatal interventions and timely newborn vaccination have helped to lower HBV infection risk where implemented [183,184]. Some reports also describe vaccine and antiviral escape due to mutations in the HBV genome, which may contribute to sustained transmission and variability in prevalence [96,183]. In clinical populations, the prevalence of anti-HBc and HBsAg was elevated, warranting focused prevention, screening, and vaccination efforts [182]. In line with WHO, the Amazon Basin is a pocket of elevated HDV occurrence among HBsAg positive people, shaped by migration, socioeconomic conditions, timing and coverage of HBV vaccination, and differences between HDV genotypes [3]. These observations support tailored prevention and linkage to care in Amazonian contexts.

For hepatitis C, population‑based studies reported low seroprevalence, consistent with prior literature [185]. Marked heterogeneity emerged across subgroups, with substantially higher values among PWUD, other vulnerable, and clinical populations (including patients with coagulopathies). Across PWUD studies, the most consistently reported correlates of anti‑HCV positivity were older age, longer duration of drug use, and sharing of needles/syringes or other drug paraphernalia [123–125]. In these groups, sustained case finding and prompt antiviral initiation remain priorities. Most estimates were based on anti‑HCV, with HCV RNA reported in only one study. Consequently, we cannot estimate active infection, and seropositivity may reflect past, successfully treated, or spontaneously cleared infection rather than current viremia.

The lack of information on health social determinants in a significant proportion of the analyzed studies represents an important gap in understanding health inequities in viral hepatitis in Brazil. Only 44.5% reported race/ethnicity, limiting sociodemographic infection patterns identification. This finding reinforces the low inclusion of race/ethnicity data in high-impact medical journals [186]. The reasons for these omissions remain unexplained, yet such absence affects the representativeness, equity, and applicability of research findings, with direct consequences for the prioritization of public health policies. As a solution, the engagement of multiple stakeholders (policymakers, healthcare professionals, researchers, and civil society) is needed to raise awareness, establish regulations, and promote the integration of race/ethnicity data across all stages of research, alongside the development of explicit editorial guidelines on this issue in medical journals [186,187].

Considering this context, there is a pressing need to expand national studies using robust methodologies, such as cohort and longitudinal investigations, to enable a deeper understanding of viral hepatitis determinants in Brazil. Additionally, incorporating social and structural variables into epidemiological research is essential to guide intervention strategies targeting vulnerable groups. Strengthening epidemiological surveillance, combined with investments in research and innovation, can contribute to the development of equitable public health policies and to the elimination of viral hepatitis as a public health problem in the country, in alignment with regional and population-specific needs. Examples include hepatitis D screening among individuals with reactive HBsAg from the Amazon region [19], targeted hepatitis A vaccination efforts during a recent outbreak among MSM [188] and expanded access to point-of-care diagnosis and timely treatment for vulnerable populations [189], as integral components of the national hepatitis elimination strategy within SUS.

Regarding data quality**,** a simplified assessment was conducted, focusing on inclusion criteria clarity, adequate subjects’ characterization, and transparency in reporting results, particularly concerning exposure measurements reliability. While most studies demonstrated satisfactory quality across these parameters, it is important to note that we did not perform an in-depth critical appraisal according to the methodological standards recommended for systematic reviews. As a result, conclusions are subject to limitations regarding bias risk assessment.

Finally, despite standardizing extraction criteria and holding periodic discussions among reviewers to harmonize content, subjective interpretations in article analysis could not be entirely eliminated. However, measures were taken to mitigate these limitations, including regular team meetings to review extracted indicators and clarify methodological questions throughout the process and it was possible to reach an extensive scope review that will guide health managers to formulate priority actions to eliminate hepatitis, considering sociodemographic and regional aspects. It will also help to clarify the main gaps to be filled in terms of investment in research.

## Conclusion

This review provides a comprehensive overview of viral hepatitis epidemiology in Brazil, emphasizing the vulnerable populations epidemiological profile and the regional infection distribution. It represents a pioneering effort in consolidating evidence from the past decade, identifying epidemiological patterns, research gaps, and persistent challenges in hepatitis surveillance.

Building on the gaps identified, future research should prioritize strengthening evidence generation in regions with limited scientific output but high epidemiological relevance, particularly the North of Brazil, as well as expanding nationally representative studies with more robust methodological designs, including longitudinal and population-based approaches. These efforts are essential to improve the characterization of the national epidemiological profile, including transmission patterns and disease burden. In parallel, particular attention should be given to vulnerable and hard-to-reach populations, in which higher prevalence reflects underlying social and structural determinants. This requires the incorporation of analytical variables that capture these dimensions, combined with appropriate sampling strategies to better address epidemiological inequities. Finally, efforts to translate epidemiological findings into accessible reports and data systems are essential to support decision-making processes and strengthen public health policies aligned with viral hepatitis elimination goals.

## Supporting information

S1 Table Search strategies (by database with respective filters used and search date). (DOCX)

S2 Table Database hepatitis scoping review (Supporting Information). (XLSX)

S3 Table Codebook of the viral hepatitis scoping review database. (PDF)

S4 Table Preferred Reporting Items for Systematic reviews and Meta-Analyses extension for Scoping Reviews (PRISMA-ScR) Checklist. (DOCX)
