# Supplementary material for: The epidemiology of the viral hepatitis in Brazil: A scoping review
Source: PLoS One. 2026 Jul 17;21(7):e0353840. doi: 10.1371/journal.pone.0353840 (PMC13379016; doi:10.1371/journal.pone.0353840)
Supplement: S1 Table — (DOCX) [file pone.0353840.s001.docx]

**S1 Table - Search strategies (by database with respective filters used and search date)**

|  | Database: PUB MED |  |
| --- | --- | --- |
| Search | **Strategy** | **Filters (if any)** |
| Viral Hepatitis  MESH MAJOR + TEXT WORD | (((((((((((((((((((((((((((("Hepatitis, Viral, Human/epidemiology"[Majr]) OR ((Hepatitis, Viral, Human[Text Word]) AND (epidemiology[Text Word]))) OR (("Hepatitis, Viral, Human/mortality"[Majr]) OR (("Hepatitis, Viral, Human"[Text Word]) AND ("mortality"[Text Word])))) OR (("Hepatitis, Viral, Human/transmission"[Majr]) OR (("Hepatitis, Viral, Human"[Text Word]) AND ("transmission"[Text Word])))) OR ((((hepatitis a[Text Word]) AND (epidemiology[Text Word])) OR ((hepatitis a[Text Word]) AND (mortality[Text Word])) OR ((hepatitis a[Text Word]) AND (transmission[Text Word]))))) OR ((hepatitis a vaccines[MeSH Terms]) OR (hepatitis a vaccines[Text Word]))) OR ((hepatitis a virus[MeSH Terms]) OR (hepatitis a virus[Text Word]))) OR ((Hepatitis A Virus, Human[MeSH Terms]) OR (Hepatitis A Virus, Human[Text Word]))) OR (HAV[Text Word])) OR ((((hepatitis b[Text Word]) AND (epidemiology[Text Word])) OR ((hepatitis b[Text Word]) AND (mortality[Text Word])) OR ((hepatitis b[Text Word]) AND (transmission[Text Word]))))) OR ((hepatitis b virus[MeSH Terms]) OR (hepatitis b virus[Text Word]))) OR ((hepatitis b vaccines[MeSH Terms]) OR (hepatitis b vaccines[Text Word]))) OR ((hepatitis b, chronic[MeSH Terms]) OR (hepatitis b, chronic[Text Word]))) OR (Acute Hepatitis B[Text Word])) OR (HBV[Text Word])) OR ((((hepatitis c[Text Word]) AND (epidemiology[Text Word])) OR ((hepatitis c[Text Word]) AND (mortality[Text Word])) OR ((hepatitis c[Text Word]) AND (transmission[Text Word]))))) OR ((hepatitis c, chronic[MeSH Terms]) OR (hepatitis c, chronic[Text Word]))) OR (Acute Hepatitis C[Text Word])) OR ((HCV RNA[Text Word]) OR (HCV[Text Word]))) OR (HCV[Text Word])) OR ((((hepatitis d[Text Word]) AND (epidemiology[Text Word])) OR ((hepatitis d[Text Word]) AND (mortality[Text Word])) OR ((hepatitis d[Text Word]) AND (transmission[Text Word]))))) OR ((hepatitis delta virus[MeSH Terms]) OR (hepatitis delta virus[Text Word]))) OR ((hepatitis d, chronic[MeSH Terms]) OR (hepatitis d, chronic[Text Word]))) OR (Delta Superinfection[Text Word])) OR (HDV RNA[Text Word])) OR (HDV[Text Word])) OR ((((hepatitis e[Text Word]) AND (epidemiology[Text Word])) OR ((hepatitis e[Text Word]) AND (mortality[Text Word])) OR ((hepatitis e[Text Word]) AND (transmission[Text Word]))))) OR ((hepatitis e virus[MeSH Terms]) OR (hepatitis e virus[Text Word]))) OR (HEV RNA[Text Word]) |  |
| Global Burden of disease  MESH Terms + TEXT WORD | "carcinoma, hepatocellular"[MeSH Terms] OR "carcinoma hepatocellular"[Text Word] OR "liver failure, acute"[MeSH Terms] OR "liver failure acute"[Text Word] OR "liver cirrhosis"[MeSH Terms] OR "liver cirrhosis"[Text Word] OR "liver transplantation"[MeSH Terms] OR "liver transplantation"[Text Word] OR "hepatic insufficiency"[MeSH Terms] OR "hepatic insufficiency"[Text Word] OR "acute on chronic liver failure"[MeSH Terms] OR "acute on chronic liver failure"[Text Word] OR "infectious disease transmission, vertical"[MeSH Terms] OR "infectious disease transmission vertical"[Text Word] OR "disability adjusted life years"[MeSH Terms] OR "disability adjusted life years"[Text Word] OR "global burden of disease"[MeSH Terms] OR "global burden of disease"[Text Word] |  |
| General words MESH Terms  +  TEXT WORD | Brazil"[MeSH Terms] OR "Brazil"[Text Word] |  |
| SPECIFIC  MESH Terms  +  TEXT WORD | ("Humans"[MeSH Terms] OR "Humans"[Text Word]) |  |
| Epidemiology MESH Terms  +  TEXT WORD | "Epidemiology"[MeSH Terms] OR "Epidemiology"[Text Word] OR "Prevalence"[MeSH Terms] OR "Prevalence"[Text Word] OR "Incidence"[MeSH Terms] OR "Incidence"[Text Word] OR "Mortality"[MeSH Terms] OR "Mortality"[Text Word] OR "Morbidity"[MeSH Terms] OR "Morbidity"[Text Word] OR "seroepidemiologic studies"[MeSH Terms] OR "seroepidemiologic studies"[Text Word] OR "Reinfection"[MeSH Terms] OR "Reinfection"[Text Word] OR "epidemiologic studies"[MeSH Terms] OR "epidemiologic studies"[Text Word] OR "vaccination coverage"[MeSH Terms] OR "vaccination coverage"[Text Word] OR "Transmission"[Text Word] OR "viraemic rate"[Text Word] OR "therapy rate"[Text Word] OR "healing rate"[Text Word] OR "vaccination rate"[Text Word] OR "Lethality"[Text Word] |  |
| Vulnerable Populations  MESH Terms  +  TEXT WORD | ("social vulnerability"[MeSH Terms] OR "social vulnerability"[Text Word] OR "vulnerable populations"[MeSH Terms] OR "vulnerable population"[Text Word] OR "emigrants and immigrants"[MeSH Terms] OR "Emigrants"[Text Word] OR "Immigrants"[Text Word] OR "transients and migrants"[MeSH Terms] OR "Transients"[Text Word] OR "Migrants"[Text Word] OR "Refugees"[MeSH Terms] OR "Refugees"[Text Word] OR "health personnel"[MeSH Terms] OR "health personnel"[Text Word] OR "Homosexuality"[MeSH Terms] OR "Homosexuality"[Text Word] OR "Bisexuality"[MeSH Terms] OR "Bisexuality"[Text Word] OR "transgender persons"[MeSH Terms] OR "transgender people"[Text Word] OR "transplant recipients"[MeSH Terms] OR "transplant recipients"[Text Word] OR "pregnant women"[MeSH Terms] OR "pregnant women"[Text Word] OR "indigenous peoples"[MeSH Terms] OR "indigenous peoples"[Text Word] OR "substance abuse detection"[MeSH Terms] OR "substance abuse detection"[Text Word] OR "substance abuse, intravenous"[MeSH Terms] OR "intravenous substance abuse"[Text Word] OR "drug users"[MeSH Terms] OR "drug users"[Text Word] OR "minority health"[MeSH Terms] OR "minority health"[Text Word] OR "sex offenses"[MeSH Terms] OR "sex offenses"[Text Word] OR "infectious disease transmission, vertical"[MeSH Terms] OR "infectious disease transmission vertical"[Text Word] OR ("blood transfusion"[MeSH Terms] OR "blood component transfusion"[MeSH Terms]) OR "blood component transfusion"[Text Word] OR "liver diseases"[MeSH Terms] OR "liver diseases"[Text Word] OR "transgender persons"[MeSH Terms] OR "transgender persons"[Text Word] OR "sexual and gender minorities"[MeSH Terms] OR "Sexual"[Text Word] OR "gender minorities"[Text Word] OR "gender nonconforming persons"[MeSH Terms] OR "gender nonconforming persons"[Text Word] OR ("black or african american"[MeSH Terms] OR ("Black"[All Fields] AND "or"[All Fields] AND "african"[All Fields] AND "American"[All Fields]) OR "black or african american"[All Fields] OR "blacks"[All Fields] OR "black people"[MeSH Terms] OR ("Black"[All Fields] AND "People"[All Fields]) OR "black people"[All Fields] OR "Black"[All Fields] OR "blackness"[All Fields] OR "black or african american"[MeSH Terms]) OR "black american"[Text Word] OR "renal dialysis"[MeSH Terms] OR "renal dialysis"[Text Word] OR "Adolescent"[MeSH Terms] OR "Adolescent"[Text Word] OR "black people"[MeSH Terms] OR "black people"[Text Word] OR "sex workers"[MeSH Terms] OR "sex workers"[Text Word] OR "alcohol drinking"[MeSH Terms] OR "alcohol drinking"[Text Word] OR "substance related disorders"[MeSH Terms] OR "addiction substance"[Text Word] OR "pre exposure prophylaxis"[MeSH Terms] OR "pre exposure prophylaxis"[Text Word] OR "post exposure prophylaxis"[MeSH Terms] OR "post exposure prophylaxis"[Text Word] OR "accidents, occupational"[MeSH Terms] OR "accidents occupational"[Text Word] OR "Condoms"[MeSH Terms] OR "Condoms"[Text Word] OR "immunocompromised host"[MeSH Terms] OR "immunocompromised host"[Text Word] OR "Prisoners"[MeSH Terms] OR "Prisoners"[Text Word] OR "diabetes mellitus"[MeSH Terms] OR "diabetes mellitus"[Text Word] OR "Hypertension"[MeSH Terms] OR "Hypertension"[Text Word] OR "hiv infections"[MeSH Terms] OR "hiv infections"[Text Word] OR "pregnancy complications, infectious"[MeSH Terms] OR "pregnancy complications infectious"[Text Word] OR "mental disorders"[MeSH Terms] OR "mental disorders"[Text Word] OR "family planning policy"[MeSH Terms] OR "family planning policy"[Text Word] OR "substance related disorders"[MeSH Terms] OR "substance related disorders"[Text Word] OR "ill housed persons"[MeSH Terms] OR "ill housed persons"[Text Word] OR "Homeless"[Text Word] OR "Amazon"[Text Word] OR "riparian population"[Text Word] OR "household contacts"[Text Word] OR "quilombola communities"[Text Word] OR "beauty centers"[Text Word] OR "aesthetics centers"[Text Word]) |  |
| Viral Hepatitis  MESH MAJOR + TEXT WORD | ("hepatitis, viral, human"[MeSH Major Topic] OR "hepatitis viral human"[Text Word] OR "hepatitis a"[MeSH Major Topic] OR "hepatitis a"[Text Word] OR "hepatitis a vaccines"[MeSH Major Topic] OR "hepatitis a vaccines"[Text Word] OR "hepatitis a virus"[MeSH Major Topic] OR "hepatitis a virus"[Text Word] OR "hepatitis a virus, human"[MeSH Major Topic] OR "hepatitis a virus human"[Text Word] OR "HAV"[Text Word] OR "hepatitis b"[MeSH Major Topic] OR "hepatitis b"[Text Word] OR "hepatitis b virus"[MeSH Major Topic] OR "hepatitis b virus"[Text Word] OR "hepatitis b vaccines"[MeSH Major Topic] OR "hepatitis b vaccines"[Text Word] OR "hepatitis b, chronic"[MeSH Major Topic] OR "hepatitis b chronic"[Text Word] OR "acute hepatitis b"[Text Word] OR "HBV"[Text Word] OR "hepatitis c"[MeSH Major Topic] OR "hepacivirus"[MeSH Major Topic] OR "hepatitis c"[Text Word] OR "hepatitis c, chronic"[MeSH Major Topic] OR "hepatitis c chronic"[Text Word] OR "acute hepatitis c"[Text Word] OR "hcv rna"[Text Word] OR "HCV"[Text Word] OR "hepatitis d"[MeSH Major Topic] OR "hepatitis d"[Text Word] OR "hepatitis delta virus"[MeSH Major Topic] OR "hepatitis delta virus"[Text Word] OR "hepatitis d, chronic"[MeSH Major Topic] OR "hepatitis d chronic"[Text Word] OR "delta superinfection"[Text Word] OR "hdv rna"[Text Word] OR "HDV"[Text Word] OR "hepatitis e virus"[MeSH Major Topic] OR "hepatitis e virus"[Text Word] OR "hepatitis e"[MeSH Major Topic] OR "hepatitis e"[Text Word] OR "hev rna"[Text Word] OR "HEV"[Text Word]) |  |
| Global Burden of disease  MESH Terms  +  TEXT WORD | "carcinoma, hepatocellular"[MeSH Terms] OR "carcinoma hepatocellular"[Text Word] OR "liver failure, acute"[MeSH Terms] OR "liver failure acute"[Text Word] OR "liver cirrhosis"[MeSH Terms] OR "liver cirrhosis"[Text Word] OR "liver transplantation"[MeSH Terms] OR "liver transplantation"[Text Word] OR "hepatic insufficiency"[MeSH Terms] OR "hepatic insufficiency"[Text Word] OR "acute on chronic liver failure"[MeSH Terms] OR "acute on chronic liver failure"[Text Word] OR "infectious disease transmission, vertical"[MeSH Terms] OR "infectious disease transmission vertical"[Text Word] OR "disability adjusted life years"[MeSH Terms] OR "disability adjusted life years"[Text Word] OR "global burden of disease"[MeSH Terms] OR "global burden of disease"[Text Word] |  |
| General words MESH Terms  +  TEXT WORD | "Brazil"[MeSH Terms] OR "Brazil"[Text Word] |  |
| SPECIFIC  MESH Terms  +  TEXT WORD | ("Humans"[MeSH Terms] OR "Humans"[Text Word]) |  |
| Epidemiology  MESH Terms  +  TEXT WORD | "Epidemiology"[MeSH Terms] OR "Epidemiology"[Text Word] OR "Prevalence"[MeSH Terms] OR "Prevalence"[Text Word] OR "Incidence"[MeSH Terms] OR "Incidence"[Text Word] OR "Mortality"[MeSH Terms] OR "Mortality"[Text Word] OR "Morbidity"[MeSH Terms] OR "Morbidity"[Text Word] OR "seroepidemiologic studies"[MeSH Terms] OR "seroepidemiologic studies"[Text Word] OR "Reinfection"[MeSH Terms] OR "Reinfection"[Text Word] OR "epidemiologic studies"[MeSH Terms] OR "epidemiologic studies"[Text Word] OR "vaccination coverage"[MeSH Terms] OR "vaccination coverage"[Text Word] OR "Transmission"[Text Word] OR "viraemic rate"[Text Word] OR "therapy rate"[Text Word] OR "healing rate"[Text Word] OR "vaccination rate"[Text Word] OR "Lethality"[Text Word] |  |
| Vulnerable Populations #11  MESH Terms  +  TEXT WORD | ("social vulnerability"[MeSH Terms] OR "social vulnerability"[Text Word] OR "vulnerable populations"[MeSH Terms] OR "vulnerable population"[Text Word] OR "emigrants and immigrants"[MeSH Terms] OR "Emigrants"[Text Word] OR "Immigrants"[Text Word] OR "transients and migrants"[MeSH Terms] OR "Transients"[Text Word] OR "Migrants"[Text Word] OR "Refugees"[MeSH Terms] OR "Refugees"[Text Word] OR "health personnel"[MeSH Terms] OR "health personnel"[Text Word] OR "Homosexuality"[MeSH Terms] OR "Homosexuality"[Text Word] OR "Bisexuality"[MeSH Terms] OR "Bisexuality"[Text Word] OR "transgender persons"[MeSH Terms] OR "transgender people"[Text Word] OR "transplant recipients"[MeSH Terms] OR "transplant recipients"[Text Word] OR "pregnant women"[MeSH Terms] OR "pregnant women"[Text Word] OR "indigenous peoples"[MeSH Terms] OR "indigenous peoples"[Text Word] OR "substance abuse detection"[MeSH Terms] OR "substance abuse detection"[Text Word] OR "substance abuse, intravenous"[MeSH Terms] OR "intravenous substance abuse"[Text Word] OR "drug users"[MeSH Terms] OR "drug users"[Text Word] OR "minority health"[MeSH Terms] OR "minority health"[Text Word] OR "sex offenses"[MeSH Terms] OR "sex offenses"[Text Word] OR "infectious disease transmission, vertical"[MeSH Terms] OR "infectious disease transmission vertical"[Text Word] OR ("blood transfusion"[MeSH Terms] OR "blood component transfusion"[MeSH Terms]) OR "blood component transfusion"[Text Word] OR "liver diseases"[MeSH Terms] OR "liver diseases"[Text Word] OR "transgender persons"[MeSH Terms] OR "transgender persons"[Text Word] OR "sexual and gender minorities"[MeSH Terms] OR "Sexual"[Text Word] OR "gender minorities"[Text Word] OR "gender nonconforming persons"[MeSH Terms] OR "gender nonconforming persons"[Text Word] OR ("black or african american"[MeSH Terms] OR ("Black"[All Fields] AND "or"[All Fields] AND "african"[All Fields] AND "American"[All Fields]) OR "black or african american"[All Fields] OR "blacks"[All Fields] OR "black people"[MeSH Terms] OR ("Black"[All Fields] AND "People"[All Fields]) OR "black people"[All Fields] OR "Black"[All Fields] OR "blackness"[All Fields] OR "black or african american"[MeSH Terms]) OR "black american"[Text Word] OR "renal dialysis"[MeSH Terms] OR "renal dialysis"[Text Word] OR "Adolescent"[MeSH Terms] OR "Adolescent"[Text Word] OR "black people"[MeSH Terms] OR "black people"[Text Word] OR "sex workers"[MeSH Terms] OR "sex workers"[Text Word] OR "alcohol drinking"[MeSH Terms] OR "alcohol drinking"[Text Word] OR "substance related disorders"[MeSH Terms] OR "addiction substance"[Text Word] OR "pre exposure prophylaxis"[MeSH Terms] OR "pre exposure prophylaxis"[Text Word] OR "post exposure prophylaxis"[MeSH Terms] OR "post exposure prophylaxis"[Text Word] OR "accidents, occupational"[MeSH Terms] OR "accidents occupational"[Text Word] OR "Condoms"[MeSH Terms] OR "Condoms"[Text Word] OR "immunocompromised host"[MeSH Terms] OR "immunocompromised host"[Text Word] OR "Prisoners"[MeSH Terms] OR "Prisoners"[Text Word] OR "diabetes mellitus"[MeSH Terms] OR "diabetes mellitus"[Text Word] OR "Hypertension"[MeSH Terms] OR "Hypertension"[Text Word] OR "hiv infections"[MeSH Terms] OR "hiv infections"[Text Word] OR "pregnancy complications, infectious"[MeSH Terms] OR "pregnancy complications infectious"[Text Word] OR "mental disorders"[MeSH Terms] OR "mental disorders"[Text Word] OR "family planning policy"[MeSH Terms] OR "family planning policy"[Text Word] OR "substance related disorders"[MeSH Terms] OR "substance related disorders"[Text Word] OR "ill housed persons"[MeSH Terms] OR "ill housed persons"[Text Word] OR "Homeless"[Text Word] OR "Amazon"[Text Word] OR "riparian population"[Text Word] OR "household contacts"[Text Word] OR "quilombola communities"[Text Word] OR "beauty centers"[Text Word] OR "aesthetics centers"[Text Word]) |  |
| GUIDING QUESTION 1 .1 MESH Major / MESH Terms  +  TEXT WORD | (("hepatitis, viral, human"[MeSH Major Topic] OR "hepatitis viral human"[Text Word] OR "hepatitis a"[MeSH Major Topic] OR "hepatitis a"[Text Word] OR "hepatitis a vaccines"[MeSH Major Topic] OR "hepatitis a vaccines"[Text Word] OR "hepatitis a virus"[MeSH Major Topic] OR "hepatitis a virus"[Text Word] OR "hepatitis a virus, human"[MeSH Major Topic] OR "hepatitis a virus human"[Text Word] OR "HAV"[Text Word] OR "hepatitis b"[MeSH Major Topic] OR "hepatitis b"[Text Word] OR "hepatitis b virus"[MeSH Major Topic] OR "hepatitis b virus"[Text Word] OR "hepatitis b vaccines"[MeSH Major Topic] OR "hepatitis b vaccines"[Text Word] OR "hepatitis b, chronic"[MeSH Major Topic] OR "hepatitis b chronic"[Text Word] OR "acute hepatitis b"[Text Word] OR "HBV"[Text Word] OR "hepatitis c"[MeSH Major Topic] OR "hepacivirus"[MeSH Major Topic] OR "hepatitis c"[Text Word] OR "hepatitis c, chronic"[MeSH Major Topic] OR "hepatitis c chronic"[Text Word] OR "acute hepatitis c"[Text Word] OR "hcv rna"[Text Word] OR "HCV"[Text Word] OR "hepatitis d"[MeSH Major Topic] OR "hepatitis d"[Text Word] OR "hepatitis delta virus"[MeSH Major Topic] OR "hepatitis delta virus"[Text Word] OR "hepatitis d, chronic"[MeSH Major Topic] OR "hepatitis d chronic"[Text Word] OR "delta superinfection"[Text Word] OR "hdv rna"[Text Word] OR "HDV"[Text Word] OR "hepatitis e virus"[MeSH Major Topic] OR "hepatitis e virus"[Text Word] OR "hepatitis e"[MeSH Major Topic] OR "hepatitis e"[Text Word] OR "hev rna"[Text Word] OR "HEV"[Text Word]) AND ("Epidemiology"[MeSH Terms] OR "Epidemiology"[Text Word] OR "Prevalence"[MeSH Terms] OR "Prevalence"[Text Word] OR "Incidence"[MeSH Terms] OR "Incidence"[Text Word] OR "Mortality"[MeSH Terms] OR "Mortality"[Text Word] OR "Morbidity"[MeSH Terms] OR "Morbidity"[Text Word] OR "seroepidemiologic studies"[MeSH Terms] OR "seroepidemiologic studies"[Text Word] OR "Reinfection"[MeSH Terms] OR "Reinfection"[Text Word] OR "epidemiologic studies"[MeSH Terms] OR "epidemiologic studies"[Text Word] OR "vaccination coverage"[MeSH Terms] OR "vaccination coverage"[Text Word] OR "Transmission"[Text Word] OR "viraemic rate"[Text Word] OR "therapy rate"[Text Word] OR "healing rate"[Text Word] OR "vaccination rate"[Text Word] OR "Lethality"[Text Word]) AND ("Brazil"[MeSH Terms] OR "Brazil"[Text Word]) AND ("Humans"[MeSH Terms] OR "Humans"[Text Word])) | 2013-2024 Abstract |
| GUIDING QUESTION 1 .1  MESH Major subheading / MESH Terms  +  TEXT WORD | ((((((((((((((((((((((((((((((("Hepatitis, Viral, Human/epidemiology"[Majr]) OR ((Hepatitis, Viral, Human[Text Word]) AND (epidemiology[Text Word]))) OR (("Hepatitis, Viral, Human/mortality"[Majr]) OR (("Hepatitis, Viral, Human"[Text Word]) AND ("mortality"[Text Word])))) OR (("Hepatitis, Viral, Human/transmission"[Majr]) OR (("Hepatitis, Viral, Human"[Text Word]) AND ("transmission"[Text Word])))) OR ((((hepatitis a[Text Word]) AND (epidemiology[Text Word])) OR ((hepatitis a[Text Word]) AND (mortality[Text Word])) OR ((hepatitis a[Text Word]) AND (transmission[Text Word]))))) OR ((hepatitis a vaccines[MeSH Terms]) OR (hepatitis a vaccines[Text Word]))) OR ((hepatitis a virus[MeSH Terms]) OR (hepatitis a virus[Text Word]))) OR ((Hepatitis A Virus, Human[MeSH Terms]) OR (Hepatitis A Virus, Human[Text Word]))) OR (HAV[Text Word])) OR ((((hepatitis b[Text Word]) AND (epidemiology[Text Word])) OR ((hepatitis b[Text Word]) AND (mortality[Text Word])) OR ((hepatitis b[Text Word]) AND (transmission[Text Word]))))) OR ((hepatitis b virus[MeSH Terms]) OR (hepatitis b virus[Text Word]))) OR ((hepatitis b vaccines[MeSH Terms]) OR (hepatitis b vaccines[Text Word]))) OR ((hepatitis b, chronic[MeSH Terms]) OR (hepatitis b, chronic[Text Word]))) OR (Acute Hepatitis B[Text Word])) OR (HBV[Text Word])) OR ((((hepatitis c[Text Word]) AND (epidemiology[Text Word])) OR ((hepatitis c[Text Word]) AND (mortality[Text Word])) OR ((hepatitis c[Text Word]) AND (transmission[Text Word]))))) OR ((hepatitis c, chronic[MeSH Terms]) OR (hepatitis c, chronic[Text Word]))) OR (Acute Hepatitis C[Text Word])) OR ((HCV RNA[Text Word]) OR (HCV[Text Word]))) OR (HCV[Text Word])) OR ((((hepatitis d[Text Word]) AND (epidemiology[Text Word])) OR ((hepatitis d[Text Word]) AND (mortality[Text Word])) OR ((hepatitis d[Text Word]) AND (transmission[Text Word]))))) OR ((hepatitis delta virus[MeSH Terms]) OR (hepatitis delta virus[Text Word]))) OR ((hepatitis d, chronic[MeSH Terms]) OR (hepatitis d, chronic[Text Word]))) OR (Delta Superinfection[Text Word])) OR (HDV RNA[Text Word])) OR (HDV[Text Word])) OR ((((hepatitis e[Text Word]) AND (epidemiology[Text Word])) OR ((hepatitis e[Text Word]) AND (mortality[Text Word])) OR ((hepatitis e[Text Word]) AND (transmission[Text Word]))))) OR ((hepatitis e virus[MeSH Terms]) OR (hepatitis e virus[Text Word]))) OR (HEV RNA[Text Word])) AND ("Epidemiology"[MeSH Terms] OR "Epidemiology"[Text Word] OR "Prevalence"[MeSH Terms] OR "Prevalence"[Text Word] OR "Incidence"[MeSH Terms] OR "Incidence"[Text Word] OR "Mortality"[MeSH Terms] OR "Mortality"[Text Word] OR "Morbidity"[MeSH Terms] OR "Morbidity"[Text Word] OR "seroepidemiologic studies"[MeSH Terms] OR "seroepidemiologic studies"[Text Word] OR "Reinfection"[MeSH Terms] OR "Reinfection"[Text Word] OR "epidemiologic studies"[MeSH Terms] OR "epidemiologic studies"[Text Word] OR "vaccination coverage"[MeSH Terms] OR "vaccination coverage"[Text Word] OR "Transmission"[Text Word] OR "viraemic rate"[Text Word] OR "therapy rate"[Text Word] OR "healing rate"[Text Word] OR "vaccination rate"[Text Word] OR "Lethality"[Text Word])) AND ("Brazil"[MeSH Terms] OR "Brazil"[Text Word])) AND (("Humans"[MeSH Terms] OR "Humans"[Text Word])) | 2013-2024 Abstract |
| GUIDING QUESTION 1 .2 MESH Major / MESH Terms  +  TEXT WORD | (("hepatitis, viral, human"[MeSH Major Topic] OR "hepatitis viral human"[Text Word] OR "hepatitis a"[MeSH Major Topic] OR "hepatitis a"[Text Word] OR "hepatitis a vaccines"[MeSH Major Topic] OR "hepatitis a vaccines"[Text Word] OR "hepatitis a virus"[MeSH Major Topic] OR "hepatitis a virus"[Text Word] OR "hepatitis a virus, human"[MeSH Major Topic] OR "hepatitis a virus human"[Text Word] OR "HAV"[Text Word] OR "hepatitis b"[MeSH Major Topic] OR "hepatitis b"[Text Word] OR "hepatitis b virus"[MeSH Major Topic] OR "hepatitis b virus"[Text Word] OR "hepatitis b vaccines"[MeSH Major Topic] OR "hepatitis b vaccines"[Text Word] OR "hepatitis b, chronic"[MeSH Major Topic] OR "hepatitis b chronic"[Text Word] OR "acute hepatitis b"[Text Word] OR "HBV"[Text Word] OR "hepatitis c"[MeSH Major Topic] OR "hepacivirus"[MeSH Major Topic] OR "hepatitis c"[Text Word] OR "hepatitis c, chronic"[MeSH Major Topic] OR "hepatitis c chronic"[Text Word] OR "acute hepatitis c"[Text Word] OR "hcv rna"[Text Word] OR "HCV"[Text Word] OR "hepatitis d"[MeSH Major Topic] OR "hepatitis d"[Text Word] OR "hepatitis delta virus"[MeSH Major Topic] OR "hepatitis delta virus"[Text Word] OR "hepatitis d, chronic"[MeSH Major Topic] OR "hepatitis d chronic"[Text Word] OR "delta superinfection"[Text Word] OR "hdv rna"[Text Word] OR "HDV"[Text Word] OR "hepatitis e virus"[MeSH Major Topic] OR "hepatitis e virus"[Text Word] OR "hepatitis e"[MeSH Major Topic] OR "hepatitis e"[Text Word] OR "hev rna"[Text Word] OR "HEV"[Text Word]) AND ("carcinoma, hepatocellular"[MeSH Terms] OR "carcinoma hepatocellular"[Text Word] OR "liver failure, acute"[MeSH Terms] OR "liver failure acute"[Text Word] OR "liver cirrhosis"[MeSH Terms] OR "liver cirrhosis"[Text Word] OR "liver transplantation"[MeSH Terms] OR "liver transplantation"[Text Word] OR "hepatic insufficiency"[MeSH Terms] OR "hepatic insufficiency"[Text Word] OR "acute on chronic liver failure"[MeSH Terms] OR "acute on chronic liver failure"[Text Word] OR "infectious disease transmission, vertical"[MeSH Terms] OR "infectious disease transmission vertical"[Text Word] OR "disability adjusted life years"[MeSH Terms] OR "disability adjusted life year"[Text Word] OR "global burden of disease"[MeSH Terms] OR "global burden of disease"[Text Word]) AND ("Epidemiology"[MeSH Terms] OR "Epidemiology"[Text Word] OR "Prevalence"[MeSH Terms] OR "Prevalence"[Text Word] OR "Incidence"[MeSH Terms] OR "Incidence"[Text Word] OR "Mortality"[MeSH Terms] OR "Mortality"[Text Word] OR "Morbidity"[MeSH Terms] OR "Morbidity"[Text Word] OR "seroepidemiologic studies"[MeSH Terms] OR "seroepidemiologic studies"[Text Word] OR "Reinfection"[MeSH Terms] OR "Reinfection"[Text Word] OR "epidemiologic studies"[MeSH Terms] OR "epidemiologic studies"[Text Word] OR "vaccination coverage"[MeSH Terms] OR "vaccination coverage"[Text Word] OR "Transmission"[Text Word] OR "viraemic rate"[Text Word] OR "therapy rate"[Text Word] OR "healing rate"[Text Word] OR "vaccination rate"[Text Word] OR "Lethality"[Text Word]) AND ("Brazil"[MeSH Terms] OR "Brazil"[Text Word]) AND ("Humans"[MeSH Terms] OR "Humans"[Text Word])) | 2013-2024  Abstract |
| GUIDING QUESTION 1 .2  MESH Major subheading / MESH Terms  +  TEXT WORD | (((((((((((((((((((((((((((((((("Hepatitis, Viral, Human/epidemiology"[Majr]) OR ((Hepatitis, Viral, Human[Text Word]) AND (epidemiology[Text Word]))) OR (("Hepatitis, Viral, Human/mortality"[Majr]) OR (("Hepatitis, Viral, Human"[Text Word]) AND ("mortality"[Text Word])))) OR (("Hepatitis, Viral, Human/transmission"[Majr]) OR (("Hepatitis, Viral, Human"[Text Word]) AND ("transmission"[Text Word])))) OR ((((hepatitis a[Text Word]) AND (epidemiology[Text Word])) OR ((hepatitis a[Text Word]) AND (mortality[Text Word])) OR ((hepatitis a[Text Word]) AND (transmission[Text Word]))))) OR ((hepatitis a vaccines[MeSH Terms]) OR (hepatitis a vaccines[Text Word]))) OR ((hepatitis a virus[MeSH Terms]) OR (hepatitis a virus[Text Word]))) OR ((Hepatitis A Virus, Human[MeSH Terms]) OR (Hepatitis A Virus, Human[Text Word]))) OR (HAV[Text Word])) OR ((((hepatitis b[Text Word]) AND (epidemiology[Text Word])) OR ((hepatitis b[Text Word]) AND (mortality[Text Word])) OR ((hepatitis b[Text Word]) AND (transmission[Text Word]))))) OR ((hepatitis b virus[MeSH Terms]) OR (hepatitis b virus[Text Word]))) OR ((hepatitis b vaccines[MeSH Terms]) OR (hepatitis b vaccines[Text Word]))) OR ((hepatitis b, chronic[MeSH Terms]) OR (hepatitis b, chronic[Text Word]))) OR (Acute Hepatitis B[Text Word])) OR (HBV[Text Word])) OR ((((hepatitis c[Text Word]) AND (epidemiology[Text Word])) OR ((hepatitis c[Text Word]) AND (mortality[Text Word])) OR ((hepatitis c[Text Word]) AND (transmission[Text Word]))))) OR ((hepatitis c, chronic[MeSH Terms]) OR (hepatitis c, chronic[Text Word]))) OR (Acute Hepatitis C[Text Word])) OR ((HCV RNA[Text Word]) OR (HCV[Text Word]))) OR (HCV[Text Word])) OR ((((hepatitis d[Text Word]) AND (epidemiology[Text Word])) OR ((hepatitis d[Text Word]) AND (mortality[Text Word])) OR ((hepatitis d[Text Word]) AND (transmission[Text Word]))))) OR ((hepatitis delta virus[MeSH Terms]) OR (hepatitis delta virus[Text Word]))) OR ((hepatitis d, chronic[MeSH Terms]) OR (hepatitis d, chronic[Text Word]))) OR (Delta Superinfection[Text Word])) OR (HDV RNA[Text Word])) OR (HDV[Text Word])) OR ((((hepatitis e[Text Word]) AND (epidemiology[Text Word])) OR ((hepatitis e[Text Word]) AND (mortality[Text Word])) OR ((hepatitis e[Text Word]) AND (transmission[Text Word]))))) OR ((hepatitis e virus[MeSH Terms]) OR (hepatitis e virus[Text Word]))) OR (HEV RNA[Text Word])) AND ("carcinoma, hepatocellular"[MeSH Terms] OR "carcinoma hepatocellular"[Text Word] OR "liver failure, acute"[MeSH Terms] OR "liver failure acute"[Text Word] OR "liver cirrhosis"[MeSH Terms] OR "liver cirrhosis"[Text Word] OR "liver transplantation"[MeSH Terms] OR "liver transplantation"[Text Word] OR "hepatic insufficiency"[MeSH Terms] OR "hepatic insufficiency"[Text Word] OR "acute on chronic liver failure"[MeSH Terms] OR "acute on chronic liver failure"[Text Word] OR "infectious disease transmission, vertical"[MeSH Terms] OR "infectious disease transmission vertical"[Text Word] OR "disability adjusted life years"[MeSH Terms] OR "disability adjusted life years"[Text Word] OR "global burden of disease"[MeSH Terms] OR "global burden of disease"[Text Word])) AND ("Epidemiology"[MeSH Terms] OR "Epidemiology"[Text Word] OR "Prevalence"[MeSH Terms] OR "Prevalence"[Text Word] OR "Incidence"[MeSH Terms] OR "Incidence"[Text Word] OR "Mortality"[MeSH Terms] OR "Mortality"[Text Word] OR "Morbidity"[MeSH Terms] OR "Morbidity"[Text Word] OR "seroepidemiologic studies"[MeSH Terms] OR "seroepidemiologic studies"[Text Word] OR "Reinfection"[MeSH Terms] OR "Reinfection"[Text Word] OR "epidemiologic studies"[MeSH Terms] OR "epidemiologic studies"[Text Word] OR "vaccination coverage"[MeSH Terms] OR "vaccination coverage"[Text Word] OR "Transmission"[Text Word] OR "viraemic rate"[Text Word] OR "therapy rate"[Text Word] OR "healing rate"[Text Word] OR "vaccination rate"[Text Word] OR "Lethality"[Text Word])) AND ("Brazil"[MeSH Terms] OR "Brazil"[Text Word])) AND (("Humans"[MeSH Terms] OR "Humans"[Text Word])) | 2013-2024 Abstract |
| GUIDING QUESTION 1 .3 MESH Major / MESH Terms  +  TEXT WORD | (("hepatitis, viral, human"[MeSH Major Topic] OR "hepatitis viral human"[Text Word] OR "hepatitis a"[MeSH Major Topic] OR "hepatitis a"[Text Word] OR "hepatitis a vaccines"[MeSH Major Topic] OR "hepatitis a vaccines"[Text Word] OR "hepatitis a virus"[MeSH Major Topic] OR "hepatitis a virus"[Text Word] OR "hepatitis a virus, human"[MeSH Major Topic] OR "hepatitis a virus human"[Text Word] OR "HAV"[Text Word] OR "hepatitis b"[MeSH Major Topic] OR "hepatitis b"[Text Word] OR "hepatitis b virus"[MeSH Major Topic] OR "hepatitis b virus"[Text Word] OR "hepatitis b vaccines"[MeSH Major Topic] OR "hepatitis b vaccines"[Text Word] OR "hepatitis b, chronic"[MeSH Major Topic] OR "hepatitis b chronic"[Text Word] OR "acute hepatitis b"[Text Word] OR "HBV"[Text Word] OR "hepatitis c"[MeSH Major Topic] OR "hepacivirus"[MeSH Major Topic] OR "hepatitis c"[Text Word] OR "hepatitis c, chronic"[MeSH Major Topic] OR "hepatitis c chronic"[Text Word] OR "acute hepatitis c"[Text Word] OR "hcv rna"[Text Word] OR "HCV"[Text Word] OR "hepatitis d"[MeSH Major Topic] OR "hepatitis d"[Text Word] OR "hepatitis delta virus"[MeSH Major Topic] OR "hepatitis delta virus"[Text Word] OR "hepatitis d, chronic"[MeSH Major Topic] OR "hepatitis d chronic"[Text Word] OR "delta superinfection"[Text Word] OR "hdv rna"[Text Word] OR "HDV"[Text Word] OR "hepatitis e virus"[MeSH Major Topic] OR "hepatitis e virus"[Text Word] OR "hepatitis e"[MeSH Major Topic] OR "hepatitis e"[Text Word] OR "hev rna"[Text Word] OR "HEV"[Text Word]) AND ("Epidemiology"[MeSH Terms] OR "Epidemiology"[Text Word] OR "Prevalence"[MeSH Terms] OR "Prevalence"[Text Word] OR "Incidence"[MeSH Terms] OR "Incidence"[Text Word] OR "Mortality"[MeSH Terms] OR "Mortality"[Text Word] OR "Morbidity"[MeSH Terms] OR "Morbidity"[Text Word] OR "seroepidemiologic studies"[MeSH Terms] OR "seroepidemiologic studies"[Text Word] OR "Reinfection"[MeSH Terms] OR "Reinfection"[Text Word] OR "epidemiologic studies"[MeSH Terms] OR "epidemiologic studies"[Text Word] OR "vaccination coverage"[MeSH Terms] OR "vaccination coverage"[Text Word] OR "Transmission"[Text Word] OR "viraemic rate"[Text Word] OR "therapy rate"[Text Word] OR "healing rate"[Text Word] OR "vaccination rate"[Text Word] OR "Lethality"[Text Word]) AND ("social vulnerability"[MeSH Terms] OR "social vulnerability"[Text Word] OR "vulnerable populations"[MeSH Terms] OR "vulnerable population"[Text Word] OR "emigrants and immigrants"[MeSH Terms] OR "Emigrants"[Text Word] OR "Immigrants"[Text Word] OR "transients and migrants"[MeSH Terms] OR "Transients"[Text Word] OR "Migrants"[Text Word] OR "Refugees"[MeSH Terms] OR "Refugees"[Text Word] OR "health personnel"[MeSH Terms] OR "health personnel"[Text Word] OR "Homosexuality"[MeSH Terms] OR "Homosexuality"[Text Word] OR "Bisexuality"[MeSH Terms] OR "Bisexuality"[Text Word] OR "transgender persons"[MeSH Terms] OR "transgender people"[Text Word] OR "transplant recipients"[MeSH Terms] OR "transplant recipients"[Text Word] OR "pregnant women"[MeSH Terms] OR "pregnant women"[Text Word] OR "indigenous peoples"[MeSH Terms] OR "indigenous peoples"[Text Word] OR "substance abuse detection"[MeSH Terms] OR "substance abuse detection"[Text Word] OR "substance abuse, intravenous"[MeSH Terms] OR "intravenous substance abuse"[Text Word] OR "drug users"[MeSH Terms] OR "drug users"[Text Word] OR "minority health"[MeSH Terms] OR "minority health"[Text Word] OR "sex offenses"[MeSH Terms] OR "sex offenses"[Text Word] OR "infectious disease transmission, vertical"[MeSH Terms] OR "infectious disease transmission vertical"[Text Word] OR ("blood transfusion"[MeSH Terms] OR "blood component transfusion"[MeSH Terms]) OR "blood component transfusion"[Text Word] OR "liver diseases"[MeSH Terms] OR "liver diseases"[Text Word] OR "transgender persons"[MeSH Terms] OR "transgender persons"[Text Word] OR "sexual and gender minorities"[MeSH Terms] OR "Sexual"[Text Word] OR "gender minorities"[Text Word] OR "gender nonconforming persons"[MeSH Terms] OR "gender nonconforming persons"[Text Word] OR ("black or african american"[MeSH Terms] OR ("Black"[All Fields] AND "or"[All Fields] AND "african"[All Fields] AND "American"[All Fields]) OR "black or african american"[All Fields] OR "blacks"[All Fields] OR "black people"[MeSH Terms] OR ("Black"[All Fields] AND "People"[All Fields]) OR "black people"[All Fields] OR "Black"[All Fields] OR "blackness"[All Fields] OR "black or african american"[MeSH Terms]) OR "black american"[Text Word] OR "renal dialysis"[MeSH Terms] OR "renal dialysis"[Text Word] OR "Adolescent"[MeSH Terms] OR "Adolescent"[Text Word] OR "black people"[MeSH Terms] OR "black people"[Text Word] OR "sex workers"[MeSH Terms] OR "sex workers"[Text Word] OR "alcohol drinking"[MeSH Terms] OR "alcohol drinking"[Text Word] OR "substance related disorders"[MeSH Terms] OR "addiction substance"[Text Word] OR "pre exposure prophylaxis"[MeSH Terms] OR "pre exposure prophylaxis"[Text Word] OR "post exposure prophylaxis"[MeSH Terms] OR "post exposure prophylaxis"[Text Word] OR "accidents, occupational"[MeSH Terms] OR "accidents occupational"[Text Word] OR "Condoms"[MeSH Terms] OR "Condoms"[Text Word] OR "immunocompromised host"[MeSH Terms] OR "immunocompromised host"[Text Word] OR "Prisoners"[MeSH Terms] OR "Prisoners"[Text Word] OR "diabetes mellitus"[MeSH Terms] OR "diabetes mellitus"[Text Word] OR "Hypertension"[MeSH Terms] OR "Hypertension"[Text Word] OR "hiv infections"[MeSH Terms] OR "hiv infections"[Text Word] OR "pregnancy complications, infectious"[MeSH Terms] OR "pregnancy complications infectious"[Text Word] OR "mental disorders"[MeSH Terms] OR "mental disorders"[Text Word] OR "family planning policy"[MeSH Terms] OR "family planning policy"[Text Word] OR "substance related disorders"[MeSH Terms] OR "substance related disorders"[Text Word] OR "ill housed persons"[MeSH Terms] OR "ill housed persons"[Text Word] OR "Homeless"[Text Word] OR "Amazon"[Text Word] OR "riparian population"[Text Word] OR "household contacts"[Text Word] OR "quilombola communities"[Text Word] OR "beauty centers"[Text Word] OR "aesthetics centers"[Text Word]) AND ("Brazil"[MeSH Terms] OR "Brazil"[Text Word]) AND ("Humans"[MeSH Terms] OR "Humans"[Text Word])) | 2013-2024 Abstract |
| GUIDING QUESTION 1 .3  MESH Major subheading / MESH Terms  +  TEXT WORD | (((((((((((((((((((((((((((((((("Hepatitis, Viral, Human/epidemiology"[Majr]) OR ((Hepatitis, Viral, Human[Text Word]) AND (epidemiology[Text Word]))) OR (("Hepatitis, Viral, Human/mortality"[Majr]) OR (("Hepatitis, Viral, Human"[Text Word]) AND ("mortality"[Text Word])))) OR (("Hepatitis, Viral, Human/transmission"[Majr]) OR (("Hepatitis, Viral, Human"[Text Word]) AND ("transmission"[Text Word])))) OR ((((hepatitis a[Text Word]) AND (epidemiology[Text Word])) OR ((hepatitis a[Text Word]) AND (mortality[Text Word])) OR ((hepatitis a[Text Word]) AND (transmission[Text Word]))))) OR ((hepatitis a vaccines[MeSH Terms]) OR (hepatitis a vaccines[Text Word]))) OR ((hepatitis a virus[MeSH Terms]) OR (hepatitis a virus[Text Word]))) OR ((Hepatitis A Virus, Human[MeSH Terms]) OR (Hepatitis A Virus, Human[Text Word]))) OR (HAV[Text Word])) OR ((((hepatitis b[Text Word]) AND (epidemiology[Text Word])) OR ((hepatitis b[Text Word]) AND (mortality[Text Word])) OR ((hepatitis b[Text Word]) AND (transmission[Text Word]))))) OR ((hepatitis b virus[MeSH Terms]) OR (hepatitis b virus[Text Word]))) OR ((hepatitis b vaccines[MeSH Terms]) OR (hepatitis b vaccines[Text Word]))) OR ((hepatitis b, chronic[MeSH Terms]) OR (hepatitis b, chronic[Text Word]))) OR (Acute Hepatitis B[Text Word])) OR (HBV[Text Word])) OR ((((hepatitis c[Text Word]) AND (epidemiology[Text Word])) OR ((hepatitis c[Text Word]) AND (mortality[Text Word])) OR ((hepatitis c[Text Word]) AND (transmission[Text Word]))))) OR ((hepatitis c, chronic[MeSH Terms]) OR (hepatitis c, chronic[Text Word]))) OR (Acute Hepatitis C[Text Word])) OR ((HCV RNA[Text Word]) OR (HCV[Text Word]))) OR (HCV[Text Word])) OR ((((hepatitis d[Text Word]) AND (epidemiology[Text Word])) OR ((hepatitis d[Text Word]) AND (mortality[Text Word])) OR ((hepatitis d[Text Word]) AND (transmission[Text Word]))))) OR ((hepatitis delta virus[MeSH Terms]) OR (hepatitis delta virus[Text Word]))) OR ((hepatitis d, chronic[MeSH Terms]) OR (hepatitis d, chronic[Text Word]))) OR (Delta Superinfection[Text Word])) OR (HDV RNA[Text Word])) OR (HDV[Text Word])) OR ((((hepatitis e[Text Word]) AND (epidemiology[Text Word])) OR ((hepatitis e[Text Word]) AND (mortality[Text Word])) OR ((hepatitis e[Text Word]) AND (transmission[Text Word]))))) OR ((hepatitis e virus[MeSH Terms]) OR (hepatitis e virus[Text Word]))) OR (HEV RNA[Text Word])) AND ("Epidemiology"[MeSH Terms] OR "Epidemiology"[Text Word] OR "Prevalence"[MeSH Terms] OR "Prevalence"[Text Word] OR "Incidence"[MeSH Terms] OR "Incidence"[Text Word] OR "Mortality"[MeSH Terms] OR "Mortality"[Text Word] OR "Morbidity"[MeSH Terms] OR "Morbidity"[Text Word] OR "seroepidemiologic studies"[MeSH Terms] OR "seroepidemiologic studies"[Text Word] OR "Reinfection"[MeSH Terms] OR "Reinfection"[Text Word] OR "epidemiologic studies"[MeSH Terms] OR "epidemiologic studies"[Text Word] OR "vaccination coverage"[MeSH Terms] OR "vaccination coverage"[Text Word] OR "Transmission"[Text Word] OR "viraemic rate"[Text Word] OR "therapy rate"[Text Word] OR "healing rate"[Text Word] OR "vaccination rate"[Text Word] OR "Lethality"[Text Word])) AND (("social vulnerability"[MeSH Terms] OR "social vulnerability"[Text Word] OR "vulnerable populations"[MeSH Terms] OR "vulnerable population"[Text Word] OR "emigrants and immigrants"[MeSH Terms] OR "Emigrants"[Text Word] OR "Immigrants"[Text Word] OR "transients and migrants"[MeSH Terms] OR "Transients"[Text Word] OR "Migrants"[Text Word] OR "Refugees"[MeSH Terms] OR "Refugees"[Text Word] OR "health personnel"[MeSH Terms] OR "health personnel"[Text Word] OR "Homosexuality"[MeSH Terms] OR "Homosexuality"[Text Word] OR "Bisexuality"[MeSH Terms] OR "Bisexuality"[Text Word] OR "transgender persons"[MeSH Terms] OR "transgender people"[Text Word] OR "transplant recipients"[MeSH Terms] OR "transplant recipients"[Text Word] OR "pregnant women"[MeSH Terms] OR "pregnant women"[Text Word] OR "indigenous peoples"[MeSH Terms] OR "indigenous peoples"[Text Word] OR "substance abuse detection"[MeSH Terms] OR "substance abuse detection"[Text Word] OR "substance abuse, intravenous"[MeSH Terms] OR "intravenous substance abuse"[Text Word] OR "drug users"[MeSH Terms] OR "drug users"[Text Word] OR "minority health"[MeSH Terms] OR "minority health"[Text Word] OR "sex offenses"[MeSH Terms] OR "sex offenses"[Text Word] OR "infectious disease transmission, vertical"[MeSH Terms] OR "infectious disease transmission vertical"[Text Word] OR ("blood transfusion"[MeSH Terms] OR "blood component transfusion"[MeSH Terms]) OR "blood component transfusion"[Text Word] OR "liver diseases"[MeSH Terms] OR "liver diseases"[Text Word] OR "transgender persons"[MeSH Terms] OR "transgender persons"[Text Word] OR "sexual and gender minorities"[MeSH Terms] OR "Sexual"[Text Word] OR "gender minorities"[Text Word] OR "gender nonconforming persons"[MeSH Terms] OR "gender nonconforming persons"[Text Word] OR ("black or african american"[MeSH Terms] OR ("Black"[All Fields] AND "or"[All Fields] AND "african"[All Fields] AND "American"[All Fields]) OR "black or african american"[All Fields] OR "blacks"[All Fields] OR "black people"[MeSH Terms] OR ("Black"[All Fields] AND "People"[All Fields]) OR "black people"[All Fields] OR "Black"[All Fields] OR "blackness"[All Fields] OR "black or african american"[MeSH Terms]) OR "black american"[Text Word] OR "renal dialysis"[MeSH Terms] OR "renal dialysis"[Text Word] OR "Adolescent"[MeSH Terms] OR "Adolescent"[Text Word] OR "black people"[MeSH Terms] OR "black people"[Text Word] OR "sex workers"[MeSH Terms] OR "sex workers"[Text Word] OR "alcohol drinking"[MeSH Terms] OR "alcohol drinking"[Text Word] OR "substance related disorders"[MeSH Terms] OR "addiction substance"[Text Word] OR "pre exposure prophylaxis"[MeSH Terms] OR "pre exposure prophylaxis"[Text Word] OR "post exposure prophylaxis"[MeSH Terms] OR "post exposure prophylaxis"[Text Word] OR "accidents, occupational"[MeSH Terms] OR "accidents occupational"[Text Word] OR "Condoms"[MeSH Terms] OR "Condoms"[Text Word] OR "immunocompromised host"[MeSH Terms] OR "immunocompromised host"[Text Word] OR "Prisoners"[MeSH Terms] OR "Prisoners"[Text Word] OR "diabetes mellitus"[MeSH Terms] OR "diabetes mellitus"[Text Word] OR "Hypertension"[MeSH Terms] OR "Hypertension"[Text Word] OR "hiv infections"[MeSH Terms] OR "hiv infections"[Text Word] OR "pregnancy complications, infectious"[MeSH Terms] OR "pregnancy complications infectious"[Text Word] OR "mental disorders"[MeSH Terms] OR "mental disorders"[Text Word] OR "family planning policy"[MeSH Terms] OR "family planning policy"[Text Word] OR "substance related disorders"[MeSH Terms] OR "substance related disorders"[Text Word] OR "ill housed persons"[MeSH Terms] OR "ill housed persons"[Text Word] OR "Homeless"[Text Word] OR "Amazon"[Text Word] OR "riparian population"[Text Word] OR "household contacts"[Text Word] OR "quilombola communities"[Text Word] OR "beauty centers"[Text Word] OR "aesthetics centers"[Text Word]))) AND ("Brazil"[MeSH Terms] OR "Brazil"[Text Word])) AND (("Humans"[MeSH Terms] OR "Humans"[Text Word])) | 2013-2024 Abstract |

|  | Database: EMBASE |  |
| --- | --- | --- |
| Search | **Strategy** | **Filters (if any)** |
| Viral Hepatitis | ('virus hepatitis'/exp OR 'hepatitis a'/exp OR 'hepatitis a vaccine'/exp OR 'hepatitis a virus'/exp OR 'human hepatitis a virus'/exp OR 'hepatitis b'/exp OR 'hepatitis b virus'/exp OR 'hepatitis b vaccine'/exp OR 'acute hepatitis b'/exp OR 'chronic hepatitis b'/exp OR 'hepatitis c'/exp OR 'chronic hepatitis c'/exp OR 'acute hepatitis c'/exp OR 'hepatitis d'/exp OR 'hepatitis delta virus'/exp OR 'chronic hepatitis d'/exp OR 'delta superinfection'/exp OR 'hepatitis e virus'/exp OR 'hepatitis e'/exp) OR (hav:ti,ab,kw OR hbv:ti,ab,kw OR 'hcv rna':ti,ab,kw OR hcv:ti,ab,kw OR 'hdv rna':ti,ab,kw OR hdv:ti,ab,kw OR 'hev rna':ti,ab,kw OR hev:ti,ab,kw) |  |
| Global Burden | 'liver cell carcinoma'/exp OR 'acute liver failure'/exp OR 'liver cirrhosis'/exp OR 'liver transplantation'/exp OR 'liver failure'/exp OR 'acute on chronic liver failure'/exp OR 'vertical transmission'/exp OR 'disability-adjusted life year'/exp OR 'global disease burden'/exp |  |
| General words | 'Brazil'/exp |  |
| SPECIFIC | 'human'/exp |  |
| Epidemiology | epidemiology'/exp OR 'prevalence'/exp OR 'incidence'/exp OR 'mortality'/exp OR 'morbidity'/exp OR 'seroepidemiology'/exp OR 'reinfection'/exp OR 'epidemiologic studies':ti,ab,kw OR 'vaccination coverage'/exp OR 'disease transmission'/exp OR 'viraemic rate':ti,ab,kw OR 'therapy rate':ti,ab,kw OR 'healing rate'/exp OR 'vaccination rate'/exp OR 'lethality'/exp |  |
| Vulnerable Populations | 'social vulnerability'/exp OR 'vulnerable population'/exp OR 'migrant'/exp OR 'migration'/exp OR 'refugee'/exp OR 'health care personnel'/exp OR 'homosexuality'/exp OR 'bisexuality'/exp OR 'lgbt people'/exp OR 'graft recipient'/exp OR 'pregnant woman'/exp OR 'indigenous people'/exp OR 'substance abuse'/exp OR 'drug use'/exp OR 'minority health'/exp OR 'sexual crime'/exp OR 'vertical transmission'/exp OR 'blood component therapy'/exp OR 'liver disease'/exp OR 'transgender'/exp OR (sexual AND 'gender minority'/exp) OR 'gender nonbinary'/exp OR 'african american'/exp OR 'hemodialysis'/exp OR 'adolescent'/exp OR 'black person'/exp OR 'sex worker'/exp OR 'drinking behavior'/exp OR 'drug dependence'/exp OR 'pre-exposure prophylaxis'/exp OR 'post exposure prophylaxis'/exp OR 'occupational accident'/exp OR 'condom'/exp OR 'immunocompromised patient'/exp OR 'prisoner'/exp OR 'diabetes mellitus'/exp OR 'hypertension'/exp OR 'human immunodeficiency virus infection'/exp OR 'infectious pregnancy complications'/exp OR 'mental disease'/exp OR 'family planning policy'/exp OR 'homeless person'/exp OR 'homelessness'/exp OR 'amazon'/exp OR 'riparian population':ti,ab,kw OR 'household contacts':ti,ab,kw OR 'quilombola communities':ti,ab,kw OR 'beauty centers':ti,ab,kw OR 'aesthetics centers':ti,ab,kw |  |
| GUIDING QUESTION 1.1 | ('virus hepatitis'/exp OR 'hepatitis a'/exp OR 'hepatitis a vaccine'/exp OR 'hepatitis a virus'/exp OR 'human hepatitis a virus'/exp OR hav:ti,ab,kw OR 'hepatitis b'/exp OR 'hepatitis b virus'/exp OR 'hepatitis b vaccine'/exp OR 'chronic hepatitis b'/exp OR 'acute hepatitis b'/exp OR hbv:ti,ab,kw OR 'hepatitis c'/exp OR 'chronic hepatitis c'/exp OR 'acute hepatitis c'/exp OR 'hcv rna':ti,ab,kw OR hcv:ti,ab,kw OR 'hepatitis d'/exp OR 'hepatitis delta virus'/exp OR 'chronic hepatitis d'/exp OR 'delta superinfection'/exp OR 'hdv rna':ti,ab,kw OR hdv:ti,ab,kw OR 'hepatitis e virus'/exp OR 'hepatitis e'/exp OR 'hev rna':ti,ab,kw OR hev:ti,ab,kw) AND 'brazil'/exp AND 'human'/exp AND ('epidemiology'/exp OR 'prevalence'/exp OR 'incidence'/exp OR 'mortality'/exp OR 'morbidity'/exp OR 'seroepidemiology'/exp OR 'reinfection'/exp OR 'epidemiologic studies':ti,ab,kw OR 'vaccination coverage'/exp OR 'disease transmission'/exp OR 'viraemic rate':ti,ab,kw OR 'therapy rate':ti,ab,kw OR 'healing rate'/exp OR 'vaccination rate'/exp OR 'lethality'/exp) AND [abstracts]/lim AND [2013-2024]/py | 2013-2024 Abstract |
| GUIDING QUESTION 1.2 | ('virus hepatitis'/exp OR 'hepatitis a'/exp OR 'hepatitis a vaccine'/exp OR 'hepatitis a virus'/exp OR 'human hepatitis a virus'/exp OR hav:ti,ab,kw OR 'hepatitis b'/exp OR 'hepatitis b virus'/exp OR 'hepatitis b vaccine'/exp OR 'chronic hepatitis b'/exp OR 'acute hepatitis b'/exp OR hbv:ti,ab,kw OR 'hepatitis c'/exp OR 'chronic hepatitis c'/exp OR 'acute hepatitis c'/exp OR 'hcv rna':ti,ab,kw OR hcv:ti,ab,kw OR 'hepatitis d'/exp OR 'hepatitis delta virus'/exp OR 'chronic hepatitis d'/exp OR 'delta superinfection'/exp OR 'hdv rna':ti,ab,kw OR hdv:ti,ab,kw OR 'hepatitis e virus'/exp OR 'hepatitis e'/exp OR 'hev rna':ti,ab,kw OR hev:ti,ab,kw) AND ('liver cell carcinoma'/exp OR 'acute liver failure'/exp OR 'liver cirrhosis'/exp OR 'liver transplantation'/exp OR 'liver failure'/exp OR 'acute on chronic liver failure'/exp OR 'vertical transmission'/exp OR 'disability-adjusted life year'/exp OR 'global disease burden'/exp) AND 'brazil'/exp AND 'human'/exp AND ('epidemiology'/exp OR 'prevalence'/exp OR 'incidence'/exp OR 'mortality'/exp OR 'morbidity'/exp OR 'seroepidemiology'/exp OR 'reinfection'/exp OR 'epidemiologic studies':ti,ab,kw OR 'vaccination coverage'/exp OR 'disease transmission'/exp OR 'viraemic rate':ti,ab,kw OR 'therapy rate':ti,ab,kw OR 'healing rate'/exp OR 'vaccination rate'/exp OR 'lethality'/exp) AND [abstracts]/lim AND [2013-2024]/py | 2013-2024 Abstract |
| GUIDING QUESTION 1.3 | ('virus hepatitis'/exp OR 'hepatitis a'/exp OR 'hepatitis a vaccine'/exp OR 'hepatitis a virus'/exp OR 'human hepatitis a virus'/exp OR hav:ti,ab,kw OR 'hepatitis b'/exp OR 'hepatitis b virus'/exp OR 'hepatitis b vaccine'/exp OR 'chronic hepatitis b'/exp OR 'acute hepatitis b'/exp OR hbv:ti,ab,kw OR 'hepatitis c'/exp OR 'chronic hepatitis c'/exp OR 'acute hepatitis c'/exp OR 'hcv rna':ti,ab,kw OR hcv:ti,ab,kw OR 'hepatitis d'/exp OR 'hepatitis delta virus'/exp OR 'chronic hepatitis d'/exp OR 'delta superinfection'/exp OR 'hdv rna':ti,ab,kw OR hdv:ti,ab,kw OR 'hepatitis e virus'/exp OR 'hepatitis e'/exp OR 'hev rna':ti,ab,kw OR hev:ti,ab,kw) AND 'brazil'/exp AND 'human'/exp AND ('epidemiology'/exp OR 'prevalence'/exp OR 'incidence'/exp OR 'mortality'/exp OR 'morbidity'/exp OR 'seroepidemiology'/exp OR 'reinfection'/exp OR 'epidemiologic studies':ti,ab,kw OR 'vaccination coverage'/exp OR 'disease transmission'/exp OR 'viraemic rate':ti,ab,kw OR 'therapy rate':ti,ab,kw OR 'healing rate'/exp OR 'vaccination rate'/exp OR 'lethality'/exp) AND ('social vulnerability'/exp OR 'vulnerable population'/exp OR 'migrant'/exp OR 'migration'/exp OR 'refugee'/exp OR 'health care personnel'/exp OR 'homosexuality'/exp OR 'bisexuality'/exp OR 'lgbt people'/exp OR 'graft recipient'/exp OR 'pregnant woman'/exp OR 'indigenous people'/exp OR 'substance abuse'/exp OR 'drug use'/exp OR 'minority health'/exp OR 'sexual crime'/exp OR 'vertical transmission'/exp OR 'blood component therapy'/exp OR 'liver disease'/exp OR 'transgender'/exp OR (sexual AND 'gender minority'/exp) OR 'gender nonbinary'/exp OR 'african american'/exp OR 'hemodialysis'/exp OR 'adolescent'/exp OR 'black person'/exp OR 'sex worker'/exp OR 'drinking behavior'/exp OR 'drug dependence'/exp OR 'pre-exposure prophylaxis'/exp OR 'post exposure prophylaxis'/exp OR 'occupational accident'/exp OR 'condom'/exp OR 'immunocompromised patient'/exp OR 'prisoner'/exp OR 'diabetes mellitus'/exp OR 'hypertension'/exp OR 'human immunodeficiency virus infection'/exp OR 'infectious pregnancy complications'/exp OR 'mental disease'/exp OR 'family planning policy'/exp OR 'homeless person'/exp OR 'homelessness'/exp OR 'amazon'/exp OR 'riparian population':ti,ab,kw OR 'household contacts':ti,ab,kw OR 'quilombola communities':ti,ab,kw OR 'beauty centers':ti,ab,kw OR 'aesthetics centers':ti,ab,kw) AND [abstracts]/lim AND [2013-2024]/py | 2013-2024 Abstract |

|  | Database: SCOPUS |  |
| --- | --- | --- |
| Search | **Strategy** | **Filters (if any)** |
| Viral Hepatitis | TITLE-ABS-KEY(Hepatitis, Viral, Human) OR TITLE-ABS-KEY(Hepatitis A) OR TITLE-ABS-KEY(Hepatitis A Vaccines) OR TITLE-ABS-KEY(Hepatitis A virus) OR TITLE-ABS-KEY(Hepatitis A Virus, Human) OR TITLE-ABS-KEY(HAV) OR TITLE-ABS-KEY(Hepatitis B) OR TITLE-ABS-KEY(Hepatitis B virus) OR TITLE-ABS-KEY(Hepatitis B Vaccines) OR TITLE-ABS-KEY(Hepatitis B, Chronic) OR TITLE-ABS-KEY(Acute Hepatitis B) OR TITLE-ABS-KEY(HBV) OR TITLE-ABS-KEY(Hepatitis C) OR TITLE-ABS-KEY(Hepatitis C, Chronic) OR TITLE-ABS-KEY(Acute Hepatitis C) OR TITLE-ABS-KEY(HCV RNA) OR TITLE-ABS-KEY(HCV) OR TITLE-ABS-KEY(Hepatitis D) OR TITLE-ABS-KEY(Hepatitis Delta Virus) OR TITLE-ABS-KEY(Hepatitis D, Chronic) OR TITLE-ABS-KEY(Delta Superinfection) OR TITLE-ABS-KEY(HDV RNA) OR TITLE-ABS-KEY(HDV) OR TITLE-ABS-KEY(Hepatitis E virus) OR TITLE-ABS-KEY(Hepatitis E) OR TITLE-ABS-KEY(HEV RNA) OR TITLE-ABS-KEY(HEV) |  |
| Global Burden of disease | TITLE-ABS-KEY(Carcinoma, Hepatocellular) OR TITLE-ABS-KEY(Liver Failure, Acute) OR TITLE-ABS-KEY(Liver Cirrhosis) OR TITLE-ABS-KEY(Liver Transplantation) OR TITLE-ABS-KEY(Hepatic Insufficiency) OR TITLE-ABS-KEY(Acute-On-Chronic Liver Failure) OR TITLE-ABS-KEY(Infectious Disease Transmission, Vertical) OR TITLE-ABS-KEY(Disability-adjusted life years) OR TITLE-ABS-KEY(Global burden of disease) |  |
| Epidemiology | TITLE-ABS-KEY(Epidemiology) OR TITLE-ABS-KEY(Prevalence) OR TITLE-ABS-KEY(Incidence) OR TITLE-ABS-KEY(Mortality) OR TITLE-ABS-KEY(Morbidity) OR TITLE-ABS-KEY(Seroepidemiologic Studies) OR TITLE-ABS-KEY(Reinfection) OR TITLE-ABS-KEY(Epidemiologic Studies) OR TITLE-ABS-KEY(Vaccination Coverage) OR TITLE-ABS-KEY(Transmission) OR TITLE-ABS-KEY(viraemic rate) OR TITLE-ABS-KEY(therapy rate) OR TITLE-ABS-KEY(healing rate) OR TITLE-ABS-KEY(vaccination rate) OR TITLE-ABS-KEY(Lethality) TITLE-ABS-KEY(Epidemiology) OR TITLE-ABS-KEY(Prevalence) OR TITLE-ABS-KEY(Incidence) OR TITLE-ABS-KEY(Mortality) OR TITLE-ABS-KEY(Morbidity) OR TITLE-ABS-KEY(Seroepidemiologic Studies) OR TITLE-ABS-KEY(Reinfection) OR TITLE-ABS-KEY(Epidemiologic Studies) OR TITLE-ABS-KEY(Vaccination Coverage) OR TITLE-ABS-KEY(Transmission) OR TITLE-ABS-KEY(viraemic rate) OR TITLE-ABS-KEY(therapy rate) OR TITLE-ABS-KEY(healing rate) OR TITLE-ABS-KEY(vaccination rate) OR TITLE-ABS-KEY(Lethality) |  |
| General word | TITLE-ABS-KEY(Brazil) |  |
| SPECIFIC | TITLE-ABS-KEY(Human) |  |
| Vulnerable Populations | TITLE-ABS-KEY(Social vulnerability) OR TITLE-ABS-KEY(Vulnerable population) OR TITLE-ABS-KEY(Emigrants and Immigrants) OR TITLE-ABS-KEY(Transients and Migrants) OR TITLE-ABS-KEY(Refugees) OR TITLE-ABS-KEY(Health Personnel) OR TITLE-ABS-KEY(Homosexuality) OR TITLE-ABS-KEY(Bisexuality) OR TITLE-ABS-KEY(Transgender people) OR TITLE-ABS-KEY(Transplant recipients) OR TITLE-ABS-KEY(Pregnant Women) OR TITLE-ABS-KEY(Indigenous Peoples) OR TITLE-ABS-KEY(Substance Abuse Detection) OR TITLE-ABS-KEY(Intravenous Substance Abuse) OR TITLE-ABS-KEY(Drug users) OR TITLE-ABS-KEY(Minority Health) OR TITLE-ABS-KEY(Sex Offenses) OR TITLE-ABS-KEY(Infectious Disease Transmission, Vertical) OR TITLE-ABS-KEY(Blood Component Transfusion) OR TITLE-ABS-KEY(Liver Diseases) OR TITLE-ABS-KEY(Transgender Persons) OR TITLE-ABS-KEY(Sexual and Gender Minorities) OR TITLE-ABS-KEY(Gender-Nonconforming Persons) OR TITLE-ABS-KEY(Black or African American) OR TITLE-ABS-KEY(Renal Dialysis) OR TITLE-ABS-KEY(Adolescent) OR TITLE-ABS-KEY(Black People) TITLE-ABS-KEY(Sex workers) OR TITLE-ABS-KEY(Alcohol Drinking) OR TITLE-ABS-KEY(Addiction, Substance) OR TITLE-ABS-KEY(Pre-Exposure Prophylaxis) OR TITLE-ABS-KEY(Post-Exposure Prophylaxis) OR TITLE-ABS-KEY(Accidents, Occupational) OR TITLE-ABS-KEY(Condoms) OR TITLE-ABS-KEY(Immunocompromised Host) OR TITLE-ABS-KEY(Prisoners) OR TITLE-ABS-KEY(Diabetes Mellitus) OR TITLE-ABS-KEY(Hypertension) OR TITLE-ABS-KEY(HIV Infections) OR TITLE-ABS-KEY(Pregnancy Complications, Infectious) OR TITLE-ABS-KEY(Mental Disorders) OR TITLE-ABS-KEY(Family Planning Policy) OR TITLE-ABS-KEY(Substance-Related Disorders) OR TITLE-ABS-KEY(Ill-Housed Persons) OR TITLE-ABS-KEY(Homeless) OR TITLE-ABS-KEY(Amazon) OR TITLE-ABS-KEY(Riparian population) OR TITLE-ABS-KEY(Household contacts) OR TITLE-ABS-KEY(Quilombola Communities) OR TITLE-ABS-KEY(Beauty Centers) OR TITLE-ABS-KEY(Aesthetics Centers) |  |
| GUIDING QUESTION 1.1 | (TITLE-ABS-KEY(Hepatitis, Viral, Human) OR TITLE-ABS-KEY(Hepatitis A) OR TITLE-ABS-KEY(Hepatitis A Vaccines) OR TITLE-ABS-KEY(Hepatitis A virus) OR TITLE-ABS-KEY(Hepatitis A Virus, Human) OR TITLE-ABS-KEY(HAV) OR TITLE-ABS-KEY(Hepatitis B) OR TITLE-ABS-KEY(Hepatitis B virus) OR TITLE-ABS-KEY(Hepatitis B Vaccines) OR TITLE-ABS-KEY(Hepatitis B, Chronic) OR TITLE-ABS-KEY(Acute Hepatitis B) OR TITLE-ABS-KEY(HBV) OR TITLE-ABS-KEY(Hepatitis C) OR TITLE-ABS-KEY(Hepatitis C, Chronic) OR TITLE-ABS-KEY(Acute Hepatitis C) OR TITLE-ABS-KEY(HCV RNA) OR TITLE-ABS-KEY(HCV) OR TITLE-ABS-KEY(Hepatitis D) OR TITLE-ABS-KEY(Hepatitis Delta Virus) OR TITLE-ABS-KEY(Hepatitis D, Chronic) OR TITLE-ABS-KEY(Delta Superinfection) OR TITLE-ABS-KEY(HDV RNA) OR TITLE-ABS-KEY(HDV) OR TITLE-ABS-KEY(Hepatitis E virus) OR TITLE-ABS-KEY(Hepatitis E) OR TITLE-ABS-KEY(HEV RNA) OR TITLE-ABS-KEY(HEV)) AND (TITLE-ABS-KEY(Epidemiology) OR TITLE-ABS-KEY(Prevalence) OR TITLE-ABS-KEY(Incidence) OR TITLE-ABS-KEY(Mortality) OR TITLE-ABS-KEY(Morbidity) OR TITLE-ABS-KEY(Seroepidemiologic Studies) OR TITLE-ABS-KEY(Reinfection) OR TITLE-ABS-KEY(Epidemiologic Studies) OR TITLE-ABS-KEY(Vaccination Coverage) OR TITLE-ABS-KEY(Transmission) OR TITLE-ABS-KEY(viraemic rate) OR TITLE-ABS-KEY(therapy rate) OR TITLE-ABS-KEY(healing rate) OR TITLE-ABS-KEY(vaccination rate) OR TITLE-ABS-KEY(Lethality) TITLE-ABS-KEY(Epidemiology) OR TITLE-ABS-KEY(Prevalence) OR TITLE-ABS-KEY(Incidence) OR TITLE-ABS-KEY(Mortality) OR TITLE-ABS-KEY(Morbidity) OR TITLE-ABS-KEY(Seroepidemiologic Studies) OR TITLE-ABS-KEY(Reinfection) OR TITLE-ABS-KEY(Epidemiologic Studies) OR TITLE-ABS-KEY(Vaccination Coverage) OR TITLE-ABS-KEY(Transmission) OR TITLE-ABS-KEY(viraemic rate) OR TITLE-ABS-KEY(therapy rate) OR TITLE-ABS-KEY(healing rate) OR TITLE-ABS-KEY(vaccination rate) OR TITLE-ABS-KEY(Lethality)) AND (TITLE-ABS-KEY(Brazil)) AND (TITLE-ABS-KEY(Human)) AND NOT INDEX(Medliene) AND PUBYEAR > 2012 AND PUBYEAR < 2025 | **2013-2024 Sem Medline** |
| GUIDING QUESTION 1.2 | (TITLE-ABS-KEY(Hepatitis, Viral, Human) OR TITLE-ABS-KEY(Hepatitis A) OR TITLE-ABS-KEY(Hepatitis A Vaccines) OR TITLE-ABS-KEY(Hepatitis A virus) OR TITLE-ABS-KEY(Hepatitis A Virus, Human) OR TITLE-ABS-KEY(HAV) OR TITLE-ABS-KEY(Hepatitis B) OR TITLE-ABS-KEY(Hepatitis B virus) OR TITLE-ABS-KEY(Hepatitis B Vaccines) OR TITLE-ABS-KEY(Hepatitis B, Chronic) OR TITLE-ABS-KEY(Acute Hepatitis B) OR TITLE-ABS-KEY(HBV) OR TITLE-ABS-KEY(Hepatitis C) OR TITLE-ABS-KEY(Hepatitis C, Chronic) OR TITLE-ABS-KEY(Acute Hepatitis C) OR TITLE-ABS-KEY(HCV RNA) OR TITLE-ABS-KEY(HCV) OR TITLE-ABS-KEY(Hepatitis D) OR TITLE-ABS-KEY(Hepatitis Delta Virus) OR TITLE-ABS-KEY(Hepatitis D, Chronic) OR TITLE-ABS-KEY(Delta Superinfection) OR TITLE-ABS-KEY(HDV RNA) OR TITLE-ABS-KEY(HDV) OR TITLE-ABS-KEY(Hepatitis E virus) OR TITLE-ABS-KEY(Hepatitis E) OR TITLE-ABS-KEY(HEV RNA) OR TITLE-ABS-KEY(HEV)) AND (TITLE-ABS-KEY(Carcinoma, Hepatocellular) OR TITLE-ABS-KEY(Liver Failure, Acute) OR TITLE-ABS-KEY(Liver Cirrhosis) OR TITLE-ABS-KEY(Liver Transplantation) OR TITLE-ABS-KEY(Hepatic Insufficiency) OR TITLE-ABS-KEY(Acute-On-Chronic Liver Failure) OR TITLE-ABS-KEY(Infectious Disease Transmission, Vertical) OR TITLE-ABS-KEY(Disability-adjusted life years) OR TITLE-ABS-KEY(Global burden of disease)) AND (TITLE-ABS-KEY(Epidemiology) OR TITLE-ABS-KEY(Prevalence) OR TITLE-ABS-KEY(Incidence) OR TITLE-ABS-KEY(Mortality) OR TITLE-ABS-KEY(Morbidity) OR TITLE-ABS-KEY(Seroepidemiologic Studies) OR TITLE-ABS-KEY(Reinfection) OR TITLE-ABS-KEY(Epidemiologic Studies) OR TITLE-ABS-KEY(Vaccination Coverage) OR TITLE-ABS-KEY(Transmission) OR TITLE-ABS-KEY(viraemic rate) OR TITLE-ABS-KEY(therapy rate) OR TITLE-ABS-KEY(healing rate) OR TITLE-ABS-KEY(vaccination rate) OR TITLE-ABS-KEY(Lethality) TITLE-ABS-KEY(Epidemiology) OR TITLE-ABS-KEY(Prevalence) OR TITLE-ABS-KEY(Incidence) OR TITLE-ABS-KEY(Mortality) OR TITLE-ABS-KEY(Morbidity) OR TITLE-ABS-KEY(Seroepidemiologic Studies) OR TITLE-ABS-KEY(Reinfection) OR TITLE-ABS-KEY(Epidemiologic Studies) OR TITLE-ABS-KEY(Vaccination Coverage) OR TITLE-ABS-KEY(Transmission) OR TITLE-ABS-KEY(viraemic rate) OR TITLE-ABS-KEY(therapy rate) OR TITLE-ABS-KEY(healing rate) OR TITLE-ABS-KEY(vaccination rate) OR TITLE-ABS-KEY(Lethality)) AND (TITLE-ABS-KEY(Brazil)) AND (TITLE-ABS-KEY(Human)) AND PUBYEAR > 2012 AND PUBYEAR < 2025 AND NOT INDEX(Medline) | **2013-2024 Sem Medline** |
| GUIDING QUESTION 1.3 | (TITLE-ABS-KEY(Hepatitis, Viral, Human) OR TITLE-ABS-KEY(Hepatitis A) OR TITLE-ABS-KEY(Hepatitis A Vaccines) OR TITLE-ABS-KEY(Hepatitis A virus) OR TITLE-ABS-KEY(Hepatitis A Virus, Human) OR TITLE-ABS-KEY(HAV) OR TITLE-ABS-KEY(Hepatitis B) OR TITLE-ABS-KEY(Hepatitis B virus) OR TITLE-ABS-KEY(Hepatitis B Vaccines) OR TITLE-ABS-KEY(Hepatitis B, Chronic) OR TITLE-ABS-KEY(Acute Hepatitis B) OR TITLE-ABS-KEY(HBV) OR TITLE-ABS-KEY(Hepatitis C) OR TITLE-ABS-KEY(Hepatitis C, Chronic) OR TITLE-ABS-KEY(Acute Hepatitis C) OR TITLE-ABS-KEY(HCV RNA) OR TITLE-ABS-KEY(HCV) OR TITLE-ABS-KEY(Hepatitis D) OR TITLE-ABS-KEY(Hepatitis Delta Virus) OR TITLE-ABS-KEY(Hepatitis D, Chronic) OR TITLE-ABS-KEY(Delta Superinfection) OR TITLE-ABS-KEY(HDV RNA) OR TITLE-ABS-KEY(HDV) OR TITLE-ABS-KEY(Hepatitis E virus) OR TITLE-ABS-KEY(Hepatitis E) OR TITLE-ABS-KEY(HEV RNA) OR TITLE-ABS-KEY(HEV)) AND (TITLE-ABS-KEY(Epidemiology) OR TITLE-ABS-KEY(Prevalence) OR TITLE-ABS-KEY(Incidence) OR TITLE-ABS-KEY(Mortality) OR TITLE-ABS-KEY(Morbidity) OR TITLE-ABS-KEY(Seroepidemiologic Studies) OR TITLE-ABS-KEY(Reinfection) OR TITLE-ABS-KEY(Epidemiologic Studies) OR TITLE-ABS-KEY(Vaccination Coverage) OR TITLE-ABS-KEY(Transmission) OR TITLE-ABS-KEY(viraemic rate) OR TITLE-ABS-KEY(therapy rate) OR TITLE-ABS-KEY(healing rate) OR TITLE-ABS-KEY(vaccination rate) OR TITLE-ABS-KEY(Lethality) TITLE-ABS-KEY(Epidemiology) OR TITLE-ABS-KEY(Prevalence) OR TITLE-ABS-KEY(Incidence) OR TITLE-ABS-KEY(Mortality) OR TITLE-ABS-KEY(Morbidity) OR TITLE-ABS-KEY(Seroepidemiologic Studies) OR TITLE-ABS-KEY(Reinfection) OR TITLE-ABS-KEY(Epidemiologic Studies) OR TITLE-ABS-KEY(Vaccination Coverage) OR TITLE-ABS-KEY(Transmission) OR TITLE-ABS-KEY(viraemic rate) OR TITLE-ABS-KEY(therapy rate) OR TITLE-ABS-KEY(healing rate) OR TITLE-ABS-KEY(vaccination rate) OR TITLE-ABS-KEY(Lethality)) AND (TITLE-ABS-KEY(Social vulnerability) OR TITLE-ABS-KEY(Vulnerable population) OR TITLE-ABS-KEY(Emigrants and Immigrants) OR TITLE-ABS-KEY(Transients and Migrants) OR TITLE-ABS-KEY(Refugees) OR TITLE-ABS-KEY(Health Personnel) OR TITLE-ABS-KEY(Homosexuality) OR TITLE-ABS-KEY(Bisexuality) OR TITLE-ABS-KEY(Transgender people) OR TITLE-ABS-KEY(Transplant recipients) OR TITLE-ABS-KEY(Pregnant Women) OR TITLE-ABS-KEY(Indigenous Peoples) OR TITLE-ABS-KEY(Substance Abuse Detection) OR TITLE-ABS-KEY(Intravenous Substance Abuse) OR TITLE-ABS-KEY(Drug users) OR TITLE-ABS-KEY(Minority Health) OR TITLE-ABS-KEY(Sex Offenses) OR TITLE-ABS-KEY(Infectious Disease Transmission, Vertical) OR TITLE-ABS-KEY(Blood Component Transfusion) OR TITLE-ABS-KEY(Liver Diseases) OR TITLE-ABS-KEY(Transgender Persons) OR TITLE-ABS-KEY(Sexual and Gender Minorities) OR TITLE-ABS-KEY(Gender-Nonconforming Persons) OR TITLE-ABS-KEY(Black or African American) OR TITLE-ABS-KEY(Renal Dialysis) OR TITLE-ABS-KEY(Adolescent) OR TITLE-ABS-KEY(Black People) TITLE-ABS-KEY(Sex workers) OR TITLE-ABS-KEY(Alcohol Drinking) OR TITLE-ABS-KEY(Addiction, Substance) OR TITLE-ABS-KEY(Pre-Exposure Prophylaxis) OR TITLE-ABS-KEY(Post-Exposure Prophylaxis) OR TITLE-ABS-KEY(Accidents, Occupational) OR TITLE-ABS-KEY(Condoms) OR TITLE-ABS-KEY(Immunocompromised Host) OR TITLE-ABS-KEY(Prisoners) OR TITLE-ABS-KEY(Diabetes Mellitus) OR TITLE-ABS-KEY(Hypertension) OR TITLE-ABS-KEY(HIV Infections) OR TITLE-ABS-KEY(Pregnancy Complications, Infectious) OR TITLE-ABS-KEY(Mental Disorders) OR TITLE-ABS-KEY(Family Planning Policy) OR TITLE-ABS-KEY(Substance-Related Disorders) OR TITLE-ABS-KEY(Ill-Housed Persons) OR TITLE-ABS-KEY(Homeless) OR TITLE-ABS-KEY(Amazon) OR TITLE-ABS-KEY(Riparian population) OR TITLE-ABS-KEY(Household contacts) OR TITLE-ABS-KEY(Quilombola Communities) OR TITLE-ABS-KEY(Beauty Centers) OR TITLE-ABS-KEY(Aesthetics Centers)) AND (TITLE-ABS-KEY(Brazil)) AND (TITLE-ABS-KEY(Human)) AND PUBYEAR > 2012 AND PUBYEAR < 2025 AND NOT INDEX(Medline) | **2013-2024 Sem Medline** |

|  | Database: LILACS |  |
| --- | --- | --- |
| Search | **Strategy** | **Filters (if any)** |
| Viral Hepatitis | (hepatite viral humana) OR (hepatite a) OR (vacinas contra hepatite a) OR (vírus da hepatite a) OR (vírus da hepatite a humana) OR (hav) OR (hepatite b) OR (vírus da hepatite b) OR (vacinas contra hepatite b) OR (hepatite b crônica) OR (hepatite b aguda) OR (hbv) OR (hepatite c) OR (hepatite c crônica) OR (hepatite c aguda) OR (hcv rna) OR (hcv) OR (hepatite d) OR (vírus delta da hepatite) OR (hepatite d crônica) OR (superinfecção delta) OR (hdv rna) OR (hdv) OR (vírus da hepatite e) OR (vírus da hepatite e) OR (hev rna) OR (hev) |  |
| Global Burden of disease | (carcinoma hepatocelular) OR (falência hepática aguda) OR (cirrose hepática) OR (transplante de fígado) OR (insuficiência hepática) OR (insuficiência hepática crônica agudizada) OR (transmissão vertical de doenças infecciosas) OR (anos de vida ajustados pela incapacidade) OR (carga global da doença) |  |
| Epidemiology | (epidemiologia) OR (prevalência) OR (incidência) OR (mortalidade) OR (morbidade) OR (estudos soroepidemiológicos) OR (reinfecção) OR (estudos epidemiológicos) OR (cobertura vacinal) OR (transmissão) OR (taxa virêmica) OR (taxa de terapia) OR (taxa de cura) OR (taxa de vacinação) OR (letalidade) |  |
| General word | Brasil |  |
| SPECIFIC | humano |  |
| Vulnerable Populations | (vulnerabilidade social) OR (populações vulneráveis) OR (emigrantes e imigrantes) OR (migrantes) OR (refugiados) OR (pessoal de saúde) OR (homossexualidade) OR (bissexualidade) OR (pessoas trans) OR (transplantados) OR (gestantes) OR (povos indígenas) OR (detecção do abuso de substâncias) OR (abuso de substâncias por via intravenosa) OR (usuários de drogas) OR (saúde das minorias) OR (delitos sexuais) OR (transmissão vertical de doenças infecciosas) OR (transfusão de componentes sanguíneos) OR (hepatopatias) OR (pessoas transgênero) OR (minorias sexuais e de gênero) OR (negro ou afro-americano) OR (diálise renal) OR (adolescente) OR (população negra) OR (profissionais do sexo) OR (consumo de bebidas alcoólicas) OR (transtornos relacionados ao uso de substâncias) OR (profilaxia pré-exposição) OR (profilaxia pós-exposição) OR (acidentes de trabalho) OR (preservativos) OR (hospedeiro imunocomprometido) OR (prisioneiros) OR (diabetes mellitus) OR (hipertensão) OR (infecções por hiv) OR (complicações infecciosas na gravidez) OR (deficiência intelectual) OR (política de planejamento familiar) OR (transtornos relacionados ao uso de substâncias) OR (pessoas mal alojadas) OR (morador de rua) OR (amazonas) OR (população ribeirinha) OR (contatos domésticos) OR (comunidades quilombolas) OR (centros de beleza) OR (centros de estética) OR (pessoas que não se conformam com o gênero) |  |
| GUIDING QUESTION 1.1 | ((hepatite viral humana) OR (hepatite a) OR (vacinas contra hepatite a) OR (vírus da hepatite a) OR (vírus da hepatite a humana) OR (hav) OR (hepatite b) OR (vírus da hepatite b) OR (vacinas contra hepatite b) OR (hepatite b crônica) OR (hepatite b aguda) OR (hbv) OR (hepatite c) OR (hepatite c crônica) OR (hepatite c aguda) OR (hcv rna) OR (hcv) OR (hepatite d) OR (vírus delta da hepatite) OR (hepatite d crônica) OR (superinfecção delta) OR (hdv rna) OR (hdv) OR (vírus da hepatite e) OR (vírus da hepatite e) OR (hev rna) OR (hev) ) AND ((epidemiologia) OR (prevalência) OR (incidência) OR (mortalidade) OR (morbidade) OR (estudos soroepidemiológicos ) OR (reinfecção) OR (estudos epidemiológicos) OR (cobertura vacinal) OR (transmissão) OR (taxa virêmica) OR (taxa de terapia) OR (taxa de cura) OR (taxa de vacinação) OR (letalidade) ) AND (brasil) AND (humano) | **LILACS 2013 - 2024** |
| GUIDING QUESTION 1.2 | ((hepatite viral humana) OR (hepatite a) OR (vacinas contra hepatite a) OR (vírus da hepatite a) OR (vírus da hepatite a humana) OR (hav) OR (hepatite b) OR (vírus da hepatite b) OR (vacinas contra hepatite b) OR (hepatite b crônica) OR (hepatite b aguda) OR (hbv) OR (hepatite c) OR (hepatite c crônica) OR (hepatite c aguda) OR (hcv rna) OR (hcv) OR (hepatite d) OR (vírus delta da hepatite) OR (hepatite d crônica) OR (superinfecção delta) OR (hdv rna) OR (hdv) OR (vírus da hepatite e) OR (vírus da hepatite e) OR (hev rna) OR (hev) ) AND ((carcinoma hepatocelular) OR (falência hepática aguda) OR (cirrose hepática) OR (transplante de fígado) OR (insuficiência hepática) OR (insuficiência hepática crônica agudizada) OR (transmissão vertical de doenças infecciosas) OR (anos de vida ajustados pela incapacidade) OR (carga global da doença) ) AND ((epidemiologia) OR (prevalência) OR (incidência) OR (mortalidade) OR (morbidade) OR (estudos soroepidemiológicos ) OR (reinfecção) OR (estudos epidemiológicos) OR (cobertura vacinal) OR (transmissão) OR (taxa virêmica) OR (taxa de terapia) OR (taxa de cura) OR (taxa de vacinação) OR (letalidade) ) AND (brasil) AND (humano) | **LILACS 2013 - 2024** |
| GUIDING QUESTION 1.3 | ((hepatite viral humana) OR (hepatite a) OR (vacinas contra hepatite a) OR (vírus da hepatite a) OR (vírus da hepatite a humana) OR (hav) OR (hepatite b) OR (vírus da hepatite b) OR (vacinas contra hepatite b) OR (hepatite b crônica) OR (hepatite b aguda) OR (hbv) OR (hepatite c) OR (hepatite c crônica) OR (hepatite c aguda) OR (hcv rna) OR (hcv) OR (hepatite d) OR (vírus delta da hepatite) OR (hepatite d crônica) OR (superinfecção delta) OR (hdv rna) OR (hdv) OR (vírus da hepatite e) OR (vírus da hepatite e) OR (hev rna) OR (hev) ) AND ((epidemiologia) OR (prevalência) OR (incidência) OR (mortalidade) OR (morbidade) OR (estudos soroepidemiológicos ) OR (reinfecção) OR (estudos epidemiológicos) OR (cobertura vacinal) OR (transmissão) OR (taxa virêmica) OR (taxa de terapia) OR (taxa de cura) OR (taxa de vacinação) OR (letalidade) ) AND ((vulnerabilidade social) OR (populações vulneráveis) OR (emigrantes e imigrantes) OR (migrantes) OR (refugiados) OR (pessoal de saúde) OR (homossexualidade) OR (bissexualidade) OR (pessoas trans) OR (transplantados) OR (gestantes) OR (povos indígenas) OR (detecção do abuso de substâncias) OR (abuso de substâncias por via intravenosa) OR (usuários de drogas) OR (saúde das minorias) OR (delitos sexuais) OR (transmissão vertical de doenças infecciosas) OR (transfusão de componentes sanguíneos) OR (hepatopatias) OR (pessoas transgênero) OR (minorias sexuais e de gênero) OR (negro ou afro-americano) OR (diálise renal) OR (adolescente) OR (população negra) OR (profissionais do sexo) OR (consumo de bebidas alcoólicas) OR (transtornos relacionados ao uso de substâncias) OR (profilaxia pré-exposição) OR (profilaxia pós-exposição) OR (acidentes de trabalho) OR (preservativos) OR (hospedeiro imunocomprometido) OR (prisioneiros) OR (diabetes mellitus) OR (hipertensão) OR (infecções por hiv) OR (complicações infecciosas na gravidez) OR (deficiência intelectual) OR (política de planejamento familiar) OR (transtornos relacionados ao uso de substâncias) OR (pessoas mal alojadas) OR (morador de rua) OR (amazonas) OR (população ribeirinha) OR (contatos domésticos) OR (comunidades quilombolas) OR (centros de beleza) OR (centros de estética) OR (pessoas que não se conformam com o gênero) ) AND (brasil) AND (humano) | **LILACS 2013 - 2024** |

|  | Database: Web of Science |  |
| --- | --- | --- |
| Search | **Strategy** | **Filters (if any)** |
| Viral Hepatitis | (((((((((((((((((((((((((((TS=(Hepatitis, Viral, Human)) OR TS=(Hepatitis A)) OR TS=(Hepatitis A Vaccines)) OR TS=(Hepatitis A virus)) OR TS=(Hepatitis A Virus, Human)) OR TS=(HAV)) OR TS=(Hepatitis B)) OR TS=(Hepatitis B virus)) OR TS=(Hepatitis B Vaccines)) OR TS=(Hepatitis B, Chronic)) OR TS=(Acute Hepatitis B)) OR TS=(HBV)) OR TS=(HBV)) OR TS=(Hepatitis C)) OR TS=(Hepatitis C, Chronic)) OR TS=(Acute Hepatitis C)) OR TS=(HCV RNA)) OR TS=(HCV)) OR TS=(Hepatitis D)) OR TS=(Hepatitis Delta Virus)) OR TS=(Hepatitis D, Chronic)) OR TS=(Delta Superinfection)) OR TS=(HDV RNA)) OR TS=(HDV)) OR TS=(Hepatitis E virus)) OR TS=(Hepatitis E)) OR TS=(HEV RNA)) OR TS=(HEV) |  |
| Global Burden of disease | ((((((((TS=(Carcinoma, Hepatocellular)) OR TS=(Liver Failure, Acute)) OR TS=(Liver Cirrhosis)) OR TS=(Liver Transplantation)) OR TS=(Hepatic Insufficiency)) OR TS=(Acute-On-Chronic Liver Failure)) OR TS=(Infectious Disease Transmission, Vertical)) OR TS=(Disability-adjusted life years)) OR TS=(Global burden of disease) |  |
| Epidemiology | ((((((((((((((TS=(Epidemiology)) OR TS=(Prevalence)) OR TS=(Incidence)) OR TS=(Mortality)) OR TS=(Morbidity)) OR TS=(Seroepidemiologic Studies)) OR TS=(Reinfection)) OR TS=(Epidemiologic Studies)) OR TS=(Vaccination Coverage)) OR TS=(Transmission)) OR TS=(viraemic rate)) OR TS=(therapy rate)) OR TS=(healing rate)) OR TS=(vaccination rate)) OR TS=(Lethality) |  |
| General word | TS=(Brazil) |  |
| SPECIFIC | TS=(Humans) |  |
| Vulnerable Populations | ((((((((((((((((((((((((((((((((((((((((((((((((((TS=(Social vulnerability)) OR TS=(Vulnerable population)) OR TS=(Emigrants and Immigrants)) OR TS=(Transients and Migrants)) OR TS=(Refugees)) OR TS=(Health Personnel)) OR TS=(Homosexuality)) OR TS=(Bisexuality)) OR TS=(Transgender people)) OR TS=(Transplant recipients)) OR TS=(Pregnant Women)) OR TS=(Indigenous Peoples)) OR TS=(Substance Abuse Detection)) OR TS=(Intravenous Substance Abuse)) OR TS=(Drug users)) OR TS=(Minority Health)) OR TS=(Sex Offenses)) OR TS=(Infectious Disease Transmission, Vertical)) OR TS=(Blood Component Transfusion)) OR TS=(Liver Diseases)) OR TS=(Transgender Persons )) OR TS=(Sexual and Gender Minorities)) OR TS=(Gender-Nonconforming Persons)) OR TS=(Black or African American)) OR TS=(Renal Dialysis)) OR TS=(Adolescent)) OR TS=(Black People)) OR TS=(Sex workers)) OR TS=(Alcohol Drinking)) OR TS=(Addiction, Substance)) OR TS=(Pre-Exposure Prophylaxis)) OR TS=(Post-Exposure Prophylaxis)) OR TS=(Accidents, Occupational)) OR TS=(Condoms)) OR TS=(Immunocompromised Host)) OR TS=(Prisoners)) OR TS=(Diabetes Mellitus)) OR TS=(Hypertension)) OR TS=(HIV Infections)) OR TS=(Pregnancy Complications, Infectious)) OR TS=(Mental Disorders)) OR TS=(Family Planning Policy)) OR TS=(Substance-Related Disorders)) OR TS=(Ill-Housed Persons)) OR TS=(Homeless)) OR TS=(Amazon)) OR TS=(Riparian population)) OR TS=(Household contacts)) OR TS=(Quilombola Communities )) OR TS=(Beauty Centers)) OR TS=(Aesthetics Centers) |  |
| GUIDING QUESTION 1.1 | (((((((((((((((((((((((((((TS=(Hepatitis, Viral, Human)) OR TS=(Hepatitis A)) OR TS=(Hepatitis A Vaccines)) OR TS=(Hepatitis A virus)) OR TS=(Hepatitis A Virus, Human)) OR TS=(HAV)) OR TS=(Hepatitis B)) OR TS=(Hepatitis B virus)) OR TS=(Hepatitis B Vaccines)) OR TS=(Hepatitis B, Chronic)) OR TS=(Acute Hepatitis B)) OR TS=(HBV)) OR TS=(HBV)) OR TS=(Hepatitis C)) OR TS=(Hepatitis C, Chronic)) OR TS=(Acute Hepatitis C)) OR TS=(HCV RNA)) OR TS=(HCV)) OR TS=(Hepatitis D)) OR TS=(Hepatitis Delta Virus)) OR TS=(Hepatitis D, Chronic)) OR TS=(Delta Superinfection)) OR TS=(HDV RNA)) OR TS=(HDV)) OR TS=(Hepatitis E virus)) OR TS=(Hepatitis E)) OR TS=(HEV RNA)) OR TS=(HEV) AND ((((((((((((((TS=(Epidemiology)) OR TS=(Prevalence)) OR TS=(Incidence)) OR TS=(Mortality)) OR TS=(Morbidity)) OR TS=(Seroepidemiologic Studies)) OR TS=(Reinfection)) OR TS=(Epidemiologic Studies)) OR TS=(Vaccination Coverage)) OR TS=(Transmission)) OR TS=(viraemic rate)) OR TS=(therapy rate)) OR TS=(healing rate)) OR TS=(vaccination rate)) OR TS=(Lethality) AND TS=(Brazil) AND TS=(Humans) | **01/01/2013 – 15/03/2024** |
| GUIDING QUESTION 1.2 | (((((((((((((((((((((((((((TS=(Hepatitis, Viral, Human)) OR TS=(Hepatitis A)) OR TS=(Hepatitis A Vaccines)) OR TS=(Hepatitis A virus)) OR TS=(Hepatitis A Virus, Human)) OR TS=(HAV)) OR TS=(Hepatitis B)) OR TS=(Hepatitis B virus)) OR TS=(Hepatitis B Vaccines)) OR TS=(Hepatitis B, Chronic)) OR TS=(Acute Hepatitis B)) OR TS=(HBV)) OR TS=(HBV)) OR TS=(Hepatitis C)) OR TS=(Hepatitis C, Chronic)) OR TS=(Acute Hepatitis C)) OR TS=(HCV RNA)) OR TS=(HCV)) OR TS=(Hepatitis D)) OR TS=(Hepatitis Delta Virus)) OR TS=(Hepatitis D, Chronic)) OR TS=(Delta Superinfection)) OR TS=(HDV RNA)) OR TS=(HDV)) OR TS=(Hepatitis E virus)) OR TS=(Hepatitis E)) OR TS=(HEV RNA)) OR TS=(HEV) AND ((((((((TS=(Carcinoma, Hepatocellular)) OR TS=(Liver Failure, Acute)) OR TS=(Liver Cirrhosis)) OR TS=(Liver Transplantation)) OR TS=(Hepatic Insufficiency)) OR TS=(Acute-On-Chronic Liver Failure)) OR TS=(Infectious Disease Transmission, Vertical)) OR TS=(Disability-adjusted life years)) OR TS=(Global burden of disease) AND ((((((((((((((TS=(Epidemiology)) OR TS=(Prevalence)) OR TS=(Incidence)) OR TS=(Mortality)) OR TS=(Morbidity)) OR TS=(Seroepidemiologic Studies)) OR TS=(Reinfection)) OR TS=(Epidemiologic Studies)) OR TS=(Vaccination Coverage)) OR TS=(Transmission)) OR TS=(viraemic rate)) OR TS=(therapy rate)) OR TS=(healing rate)) OR TS=(vaccination rate)) OR TS=(Lethality) AND TS=(Brazil) AND TS=(Humans) | **01/01/2013 – 15/03/2024** |
| GUIDING QUESTION 1.3 | (((((((((((((((((((((((((((TS=(Hepatitis, Viral, Human)) OR TS=(Hepatitis A)) OR TS=(Hepatitis A Vaccines)) OR TS=(Hepatitis A virus)) OR TS=(Hepatitis A Virus, Human)) OR TS=(HAV)) OR TS=(Hepatitis B)) OR TS=(Hepatitis B virus)) OR TS=(Hepatitis B Vaccines)) OR TS=(Hepatitis B, Chronic)) OR TS=(Acute Hepatitis B)) OR TS=(HBV)) OR TS=(HBV)) OR TS=(Hepatitis C)) OR TS=(Hepatitis C, Chronic)) OR TS=(Acute Hepatitis C)) OR TS=(HCV RNA)) OR TS=(HCV)) OR TS=(Hepatitis D)) OR TS=(Hepatitis Delta Virus)) OR TS=(Hepatitis D, Chronic)) OR TS=(Delta Superinfection)) OR TS=(HDV RNA)) OR TS=(HDV)) OR TS=(Hepatitis E virus)) OR TS=(Hepatitis E)) OR TS=(HEV RNA)) OR TS=(HEV) AND ((((((((((((((TS=(Epidemiology)) OR TS=(Prevalence)) OR TS=(Incidence)) OR TS=(Mortality)) OR TS=(Morbidity)) OR TS=(Seroepidemiologic Studies)) OR TS=(Reinfection)) OR TS=(Epidemiologic Studies)) OR TS=(Vaccination Coverage)) OR TS=(Transmission)) OR TS=(viraemic rate)) OR TS=(therapy rate)) OR TS=(healing rate)) OR TS=(vaccination rate)) OR TS=(Lethality) AND ((((((((((((((((((((((((((((((((((((((((((((((((((TS=(Social vulnerability)) OR TS=(Vulnerable population)) OR TS=(Emigrants and Immigrants)) OR TS=(Transients and Migrants)) OR TS=(Refugees)) OR TS=(Health Personnel)) OR TS=(Homosexuality)) OR TS=(Bisexuality)) OR TS=(Transgender people)) OR TS=(Transplant recipients)) OR TS=(Pregnant Women)) OR TS=(Indigenous Peoples)) OR TS=(Substance Abuse Detection)) OR TS=(Intravenous Substance Abuse)) OR TS=(Drug users)) OR TS=(Minority Health)) OR TS=(Sex Offenses)) OR TS=(Infectious Disease Transmission, Vertical)) OR TS=(Blood Component Transfusion)) OR TS=(Liver Diseases)) OR TS=(Transgender Persons )) OR TS=(Sexual and Gender Minorities)) OR TS=(Gender-Nonconforming Persons)) OR TS=(Black or African American)) OR TS=(Renal Dialysis)) OR TS=(Adolescent)) OR TS=(Black People)) OR TS=(Sex workers)) OR TS=(Alcohol Drinking)) OR TS=(Addiction, Substance)) OR TS=(Pre-Exposure Prophylaxis)) OR TS=(Post-Exposure Prophylaxis)) OR TS=(Accidents, Occupational)) OR TS=(Condoms)) OR TS=(Immunocompromised Host)) OR TS=(Prisoners)) OR TS=(Diabetes Mellitus)) OR TS=(Hypertension)) OR TS=(HIV Infections)) OR TS=(Pregnancy Complications, Infectious)) OR TS=(Mental Disorders)) OR TS=(Family Planning Policy)) OR TS=(Substance-Related Disorders)) OR TS=(Ill-Housed Persons)) OR TS=(Homeless)) OR TS=(Amazon)) OR TS=(Riparian population)) OR TS=(Household contacts)) OR TS=(Quilombola Communities )) OR TS=(Beauty Centers)) OR TS=(Aesthetics Centers) AND TS=(Brazil) AND TS=(Humans) | **01/01/2013 – 15/03/2024** |

|  | Database: COCHRANE |  |
| --- | --- | --- |
| Search | **Strategy** | **Filters (if any)** |
| Viral Hepatitis | ((virus hepatitis):ti,ab,kw OR (hepatitis a):ti,ab,kw OR (hepatitis a vaccine):ti,ab,kw OR (hepatitis a virus):ti,ab,kw OR (human hepatitis a virus):ti,ab,kw OR (hav):ti,ab,kw OR (hepatitis b):ti,ab,kw OR (hepatitis b virus):ti,ab,kw OR (hepatitis b vaccine):ti,ab,kw OR (acute hepatitis b):ti,ab,kw OR (chronic hepatitis b):ti,ab,kw OR (hbv):ti,ab,kw OR (hepatitis c):ti,ab,kw OR (chronic hepatitis c):ti,ab,kw OR (acute hepatitis c):ti,ab,kw OR (hcv rna):ti,ab,kw OR (hcv):ti,ab,kw OR (hepatitis d):ti,ab,kw OR (hepatitis delta virus):ti,ab,kw OR (chronic hepatitis d):ti,ab,kw OR (delta superinfection):ti,ab,kw OR (hdv rna):ti,ab,kw OR (hdv):ti,ab,kw OR (hepatitis e virus):ti,ab,kw OR (hepatitis e):ti,ab,kw OR (hev rna):ti,ab,kw OR (hev):ti,ab,kw) |  |
| Global Burden of disease | ((liver cell carcinoma):ti,ab,kw OR (acute liver failure):ti,ab,kw OR (liver cirrhosis):ti,ab,kw OR (liver transplantation):ti,ab,kw OR (liver failure):ti,ab,kw OR (acute on chronic liver failure):ti,ab,kw OR (vertical transmission):ti,ab,kw OR (disability-adjusted life year):ti,ab,kw OR (global disease burden):ti,ab,kw) |  |
| Epidemiology | ((epidemiology):ti,ab,kw OR (prevalence):ti,ab,kw OR (incidence):ti,ab,kw OR (mortality):ti,ab,kw OR (morbidity):ti,ab,kw OR (seroepidemiology):ti,ab,kw OR (reinfection):ti,ab,kw OR (epidemiologic studies):ti,ab,kw OR (vaccination coverage):ti,ab,kw OR (disease transmission):ti,ab,kw OR (viraemic rate):ti,ab,kw OR (therapy rate):ti,ab,kw OR (healing rate):ti,ab,kw OR (vaccination rate):ti,ab,kw OR (lethality):ti,ab,kw) |  |
| General word | (Brazil):ti,ab,kw |  |
| SPECIFIC | (Humans):ti,ab,kw |  |
| Vulnerable Populations | ((social vulnerability):ti,ab,kw OR (vulnerable population):ti,ab,kw OR (migrant):ti,ab,kw OR (migration):ti,ab,kw OR (refugee):ti,ab,kw OR (health care personnel):ti,ab,kw OR (homosexuality):ti,ab,kw OR (bisexuality):ti,ab,kw OR (lgbt people):ti,ab,kw OR (graft recipient):ti,ab,kw OR (pregnant woman):ti,ab,kw OR (indigenous people):ti,ab,kw OR (substance abuse):ti,ab,kw OR (drug use):ti,ab,kw OR (minority health):ti,ab,kw OR (sexual crime):ti,ab,kw OR (vertical transmission):ti,ab,kw OR (blood component therapy):ti,ab,kw OR (liver disease):ti,ab,kw OR (transgender):ti,ab,kw OR (sexual AND gender minority):ti,ab,kw OR (gender nonbinary):ti,ab,kw OR (african american):ti,ab,kw OR (hemodialysis):ti,ab,kw OR (adolescent):ti,ab,kw OR (black person):ti,ab,kw OR (sex worker):ti,ab,kw OR (drinking behavior):ti,ab,kw OR (drug dependence):ti,ab,kw OR (pre-exposure prophylaxis):ti,ab,kw OR (post exposure prophylaxis):ti,ab,kw OR (occupational accident):ti,ab,kw OR (condom):ti,ab,kw OR (immunocompromised patient):ti,ab,kw OR (prisoner):ti,ab,kw OR (diabetes mellitus):ti,ab,kw OR (hypertension):ti,ab,kw OR (human immunodeficiency virus infection):ti,ab,kw OR (infectious pregnancy complications):ti,ab,kw OR (mental disease):ti,ab,kw OR (family planning policy):ti,ab,kw OR (homeless person):ti,ab,kw OR (homelessness):ti,ab,kw OR (amazon):ti,ab,kw OR (riparian population):ti,ab,kw OR (household contacts):ti,ab,kw OR (quilombola communities):ti,ab,kw OR (beauty centers):ti,ab,kw OR (aesthetics centers):ti,ab,kw) |  |
| GUIDING QUESTION 1.1 | ((virus hepatitis):ti,ab,kw OR (hepatitis a):ti,ab,kw OR (hepatitis a vaccine):ti,ab,kw OR (hepatitis a virus):ti,ab,kw OR (human hepatitis a virus):ti,ab,kw OR (hav):ti,ab,kw OR (hepatitis b):ti,ab,kw OR (hepatitis b virus):ti,ab,kw OR (hepatitis b vaccine):ti,ab,kw OR (acute hepatitis b):ti,ab,kw OR (chronic hepatitis b):ti,ab,kw OR (hbv):ti,ab,kw OR (hepatitis c):ti,ab,kw OR (chronic hepatitis c):ti,ab,kw OR (acute hepatitis c):ti,ab,kw OR (hcv rna):ti,ab,kw OR (hcv):ti,ab,kw OR (hepatitis d):ti,ab,kw OR (hepatitis delta virus):ti,ab,kw OR (chronic hepatitis d):ti,ab,kw OR (delta superinfection):ti,ab,kw OR (hdv rna):ti,ab,kw OR (hdv):ti,ab,kw OR (hepatitis e virus):ti,ab,kw OR (hepatitis e):ti,ab,kw OR (hev rna):ti,ab,kw OR (hev):ti,ab,kw) AND ((epidemiology):ti,ab,kw OR (prevalence):ti,ab,kw OR (incidence):ti,ab,kw OR (mortality):ti,ab,kw OR (morbidity):ti,ab,kw OR (seroepidemiology):ti,ab,kw OR (reinfection):ti,ab,kw OR (epidemiologic studies):ti,ab,kw OR (vaccination coverage):ti,ab,kw OR (disease transmission):ti,ab,kw OR (viraemic rate):ti,ab,kw OR (therapy rate):ti,ab,kw OR (healing rate):ti,ab,kw OR (vaccination rate):ti,ab,kw OR (lethality):ti,ab,kw) AND (BRAZIL) AND (HUMANS) | **2013-2024** |
| GUIDING QUESTION 1.2 | ((virus hepatitis):ti,ab,kw OR (hepatitis a):ti,ab,kw OR (hepatitis a vaccine):ti,ab,kw OR (hepatitis a virus):ti,ab,kw OR (human hepatitis a virus):ti,ab,kw OR (hav):ti,ab,kw OR (hepatitis b):ti,ab,kw OR (hepatitis b virus):ti,ab,kw OR (hepatitis b vaccine):ti,ab,kw OR (acute hepatitis b):ti,ab,kw OR (chronic hepatitis b):ti,ab,kw OR (hbv):ti,ab,kw OR (hepatitis c):ti,ab,kw OR (chronic hepatitis c):ti,ab,kw OR (acute hepatitis c):ti,ab,kw OR (hcv rna):ti,ab,kw OR (hcv):ti,ab,kw OR (hepatitis d):ti,ab,kw OR (hepatitis delta virus):ti,ab,kw OR (chronic hepatitis d):ti,ab,kw OR (delta superinfection):ti,ab,kw OR (hdv rna):ti,ab,kw OR (hdv):ti,ab,kw OR (hepatitis e virus):ti,ab,kw OR (hepatitis e):ti,ab,kw OR (hev rna):ti,ab,kw OR (hev):ti,ab,kw) AND ((liver cell carcinoma):ti,ab,kw OR (acute liver failure):ti,ab,kw OR (liver cirrhosis):ti,ab,kw OR (liver transplantation):ti,ab,kw OR (liver failure):ti,ab,kw OR (acute on chronic liver failure):ti,ab,kw OR (vertical transmission):ti,ab,kw OR (disability-adjusted life year):ti,ab,kw OR (global disease burden):ti,ab,kw) AND ((epidemiology):ti,ab,kw OR (prevalence):ti,ab,kw OR (incidence):ti,ab,kw OR (mortality):ti,ab,kw OR (morbidity):ti,ab,kw OR (seroepidemiology):ti,ab,kw OR (reinfection):ti,ab,kw OR (epidemiologic studies):ti,ab,kw OR (vaccination coverage):ti,ab,kw OR (disease transmission):ti,ab,kw OR (viraemic rate):ti,ab,kw OR (therapy rate):ti,ab,kw OR (healing rate):ti,ab,kw OR (vaccination rate):ti,ab,kw OR (lethality):ti,ab,kw) AND (BRAZIL) AND (HUMANS) | **2013-2024** |
| GUIDING QUESTION 1.3 | ((virus hepatitis):ti,ab,kw OR (hepatitis a):ti,ab,kw OR (hepatitis a vaccine):ti,ab,kw OR (hepatitis a virus):ti,ab,kw OR (human hepatitis a virus):ti,ab,kw OR (hav):ti,ab,kw OR (hepatitis b):ti,ab,kw OR (hepatitis b virus):ti,ab,kw OR (hepatitis b vaccine):ti,ab,kw OR (acute hepatitis b):ti,ab,kw OR (chronic hepatitis b):ti,ab,kw OR (hbv):ti,ab,kw OR (hepatitis c):ti,ab,kw OR (chronic hepatitis c):ti,ab,kw OR (acute hepatitis c):ti,ab,kw OR (hcv rna):ti,ab,kw OR (hcv):ti,ab,kw OR (hepatitis d):ti,ab,kw OR (hepatitis delta virus):ti,ab,kw OR (chronic hepatitis d):ti,ab,kw OR (delta superinfection):ti,ab,kw OR (hdv rna):ti,ab,kw OR (hdv):ti,ab,kw OR (hepatitis e virus):ti,ab,kw OR (hepatitis e):ti,ab,kw OR (hev rna):ti,ab,kw OR (hev):ti,ab,kw) AND ((epidemiology):ti,ab,kw OR (prevalence):ti,ab,kw OR (incidence):ti,ab,kw OR (mortality):ti,ab,kw OR (morbidity):ti,ab,kw OR (seroepidemiology):ti,ab,kw OR (reinfection):ti,ab,kw OR (epidemiologic studies):ti,ab,kw OR (vaccination coverage):ti,ab,kw OR (disease transmission):ti,ab,kw OR (viraemic rate):ti,ab,kw OR (therapy rate):ti,ab,kw OR (healing rate):ti,ab,kw OR (vaccination rate):ti,ab,kw OR (lethality):ti,ab,kw) AND ((social vulnerability):ti,ab,kw OR (vulnerable population):ti,ab,kw OR (migrant):ti,ab,kw OR (migration):ti,ab,kw OR (refugee):ti,ab,kw OR (health care personnel):ti,ab,kw OR (homosexuality):ti,ab,kw OR (bisexuality):ti,ab,kw OR (lgbt people):ti,ab,kw OR (graft recipient):ti,ab,kw OR (pregnant woman):ti,ab,kw OR (indigenous people):ti,ab,kw OR (substance abuse):ti,ab,kw OR (drug use):ti,ab,kw OR (minority health):ti,ab,kw OR (sexual crime):ti,ab,kw OR (vertical transmission):ti,ab,kw OR (blood component therapy):ti,ab,kw OR (liver disease):ti,ab,kw OR (transgender):ti,ab,kw OR (sexual AND gender minority):ti,ab,kw OR (gender nonbinary):ti,ab,kw OR (african american):ti,ab,kw OR (hemodialysis):ti,ab,kw OR (adolescent):ti,ab,kw OR (black person):ti,ab,kw OR (sex worker):ti,ab,kw OR (drinking behavior):ti,ab,kw OR (drug dependence):ti,ab,kw OR (pre-exposure prophylaxis):ti,ab,kw OR (post exposure prophylaxis):ti,ab,kw OR (occupational accident):ti,ab,kw OR (condom):ti,ab,kw OR (immunocompromised patient):ti,ab,kw OR (prisoner):ti,ab,kw OR (diabetes mellitus):ti,ab,kw OR (hypertension):ti,ab,kw OR (human immunodeficiency virus infection):ti,ab,kw OR (infectious pregnancy complications):ti,ab,kw OR (mental disease):ti,ab,kw OR (family planning policy):ti,ab,kw OR (homeless person):ti,ab,kw OR (homelessness):ti,ab,kw OR (amazon):ti,ab,kw OR (riparian population):ti,ab,kw OR (household contacts):ti,ab,kw OR (quilombola communities):ti,ab,kw OR (beauty centers):ti,ab,kw OR (aesthetics centers):ti,ab,kw) AND (BRAZIL) AND (HUMANS) | **2013-2024** |

| Database: CINAHL | | |
| --- | --- | --- |
| Search | **Strategy** | **Filters (if any)** |
| Viral Hepatitis S1 | virus hepatitis OR hepatitis a OR hepatitis a vaccine OR hepatitis a virus OR human hepatitis a virus OR hepatitis b OR hepatitis b virus OR hepatitis b vaccine OR acute hepatitis b OR chronic hepatitis b OR hepatitis c OR chronic hepatitis c OR acute hepatitis c OR hepatitis d OR hepatitis delta virus OR chronic hepatitis d OR delta superinfection OR hepatitis e virus OR hepatitis e OR hav OR hbv OR hcv rna OR hcv OR hdv rna OR hdv OR hev rna OR hev |  |
| Global Burden of disease S2 | liver cell carcinoma OR acute liver failure OR liver cirrhosis OR liver transplantation OR liver failure OR acute on chronic liver failure OR vertical transmission OR disability-adjusted life year OR global disease burden |  |
| Epidemiology S3 | epidemiology OR prevalence OR incidence OR mortality OR morbidity OR seroepidemiology OR reinfection OR epidemiologic studies OR vaccination coverage OR disease transmission OR viraemic rate OR therapy rate OR healing rate OR vaccination rate OR lethality |  |
| Vulnerable Populations S4 | social vulnerability OR vulnerable population OR migrant OR migration OR refugee OR health care personnel OR homosexuality OR bisexuality OR lgbt people OR graft recipient OR pregnant woman OR indigenous people OR substance abuse OR drug use OR minority health OR sexual crime OR vertical transmission OR blood component therapy OR liver disease OR transgender OR (sexual AND gender minority) OR gender nonbinary OR african american OR hemodialysis OR adolescent OR black person OR sex worker OR drinking behavior OR drug dependence OR pre-exposure prophylaxis OR post exposure prophylaxis OR occupational accident OR condom OR immunocompromised patient OR prisoner OR diabetes mellitus OR hypertension OR human immunodeficiency virus infection OR infectious pregnancy complications OR mental disease OR family planning policy OR homeless person OR homelessness OR amazon OR riparian population OR household contacts OR quilombola communities OR beauty centers OR aesthetics centers |  |
| General word S5 | Brazil |  |
| SPECIFIC S6 | Human |  |
| GUIDING QUESTION 1.1 | S1 AND S3 AND S5 AND S6*  virus+hepatitis+ OR +hepatitis+a+ OR +hepatitis+a+vaccine+ OR +hepatitis+a+virus+ OR +human+hepatitis+a+virus+ OR +hepatitis+b+ OR +hepatitis+b+virus+ OR +hepatitis+b+vaccine+ OR +acute+hepatitis+b+ OR +chronic+hepatitis+b+ OR +hepatitis+c+ OR +chronic+hepatitis+c+ OR +acute+hepatitis+c+ OR +hepatitis+d+ OR +hepatitis+delta+virus+ OR +chronic+hepatitis+d+ OR +delta+superinfection+ OR +hepatitis+e+virus+ OR +hepatitis+e+ OR +hav+ OR +hbv+ OR +hcv+rna+ OR +hcv+ OR +hdv+rna+ OR +hdv+ OR +hev+rna+ OR +hev)+AND+(epidemiology+ OR +prevalence+ OR +incidence+ OR +m OR tality+ OR +m OR bidity+ OR +seroepidemiology+ OR +reinfection+ OR +epidemiologic+studies+ OR +vaccination+coverage+ OR +disease+transmission+ OR +viraemic+rate+ OR +therapy+rate+ OR +healing+rate+ OR +vaccination+rate+ OR +lethality)+AND+brazil+AND+Human&cli0=AA1&clv0=Y&cli1=DT1&clv1=201301-202412&lang=pt-br&type=1&searchMode=Standard&site=ehost-live&ssl=y | **Expanders - Apply equivalent topics Search modes - Boolean / Phrase 2013-2024 with summary** |
| GUIDING QUESTION 1.2 | S1 AND S2 AND S3 AND S5 AND S6* (virus+hepatitis+ OR +hepatitis+a+ OR +hepatitis+a+vaccine+ OR +hepatitis+a+virus+ OR +human+hepatitis+a+virus+ OR +hepatitis+b+ OR +hepatitis+b+virus+ OR +hepatitis+b+vaccine+ OR +acute+hepatitis+b+ OR +chronic+hepatitis+b+ OR +hepatitis+c+ OR +chronic+hepatitis+c+ OR +acute+hepatitis+c+ OR +hepatitis+d+ OR +hepatitis+delta+virus+ OR +chronic+hepatitis+d+ OR +delta+superinfection+ OR +hepatitis+e+virus+ OR +hepatitis+e+ OR +hav+ OR +hbv+ OR +hcv+rna+ OR +hcv+ OR +hdv+rna+ OR +hdv+ OR +hev+rna+ OR +hev)+AND+(liver+cell+carcinoma+ OR +acute+liver+failure+ OR +liver+cirrhosis+ OR +liver+transplantation+ OR +liver+failure+ OR +acute+on+chronic+liver+failure+ OR +vertical+transmission+ OR +disability-adjusted+life+year+ OR +global+disease+burden)+AND+(epidemiology+ OR +prevalence+ OR +incidence+ OR +m OR tality+ OR +m OR bidity+ OR +seroepidemiology+ OR +reinfection+ OR +epidemiologic+studies+ OR +vaccination+coverage+ OR +disease+transmission+ OR +viraemic+rate+ OR +therapy+rate+ OR +healing+rate+ OR +vaccination+rate+ OR +lethality)+AND+brazil+AND+Human&lang=pt-br&type=1&searchMode=Standard&site=ehost-live&ssl=y | **Expanders - Apply equivalent topics Search modes - Boolean / Phrase 2013-2024 with summary** |
| GUIDING QUESTION 1.3 | S1 AND S3 AND S4 AND S5 AND S6* (virus+hepatitis+ OR +hepatitis+a+ OR +hepatitis+a+vaccine+ OR +hepatitis+a+virus+ OR +human+hepatitis+a+virus+ OR +hepatitis+b+ OR +hepatitis+b+virus+ OR +hepatitis+b+vaccine+ OR +acute+hepatitis+b+ OR +chronic+hepatitis+b+ OR +hepatitis+c+ OR +chronic+hepatitis+c+ OR +acute+hepatitis+c+ OR +hepatitis+d+ OR +hepatitis+delta+virus+ OR +chronic+hepatitis+d+ OR +delta+superinfection+ OR +hepatitis+e+virus+ OR +hepatitis+e+ OR +hav+ OR +hbv+ OR +hcv+rna+ OR +hcv+ OR +hdv+rna+ OR +hdv+ OR +hev+rna+ OR +hev)+AND+(epidemiology+ OR +prevalence+ OR +incidence+ OR +m OR tality+ OR +m OR bidity+ OR +seroepidemiology+ OR +reinfection+ OR +epidemiologic+studies+ OR +vaccination+coverage+ OR +disease+transmission+ OR +viraemic+rate+ OR +therapy+rate+ OR +healing+rate+ OR +vaccination+rate+ OR +lethality)+AND+(social+vulnerability+ OR +vulnerable+population+ OR +migrant+ OR +migration+ OR +refugee+ OR +health+care+personnel+ OR +homosexuality+ OR +bisexuality+ OR +lgbt+people+ OR +graft+recipient+ OR +pregnant+woman+ OR +indigenous+people+ OR +substance+abuse+ OR +drug+use+ OR +min OR ity+health+ OR +sexual+crime+ OR +vertical+transmission+ OR +blood+component+therapy+ OR +liver+disease+ OR +transgender+ OR +(sexual+AND+gender+min OR ity)+ OR +gender+nonbinary+ OR +african+american+ OR +hemodialysis+ OR +adolescent+ OR +black+person+ OR +sex+w OR ker+ OR +drinking+behavi OR + OR +drug+dependence+ OR +pre-exposure+prophylaxis+ OR +post+exposure+prophylaxis+ OR +occupational+accident+ OR +condom+ OR +immunocompromised+patient+ OR +prisoner+ OR +diabetes+mellitus+ OR +hypertension+ OR +human+immunodeficiency+virus+infection+ OR +infectious+pregnancy+complications+ OR +mental+disease+ OR +family+planning+policy+ OR +homeless+person+ OR +homelessness+ OR +amazon+ OR +riparian+population+ OR +household+contacts+ OR +quilombola+communities+ OR +beauty+centers+ OR +aesthetics+centers)+AND+brazil+AND+Human&lang=pt-br&type=1&searchMode=Standard&site=ehost-live&ssl=y | **Expanders - Apply equivalent topics Search modes - Boolean / Phrase 2013-2024 with summary** |

* There is no space before and after the 'OR'; it was placed this way to fit better in the table.
