# Supplementary material for: The epidemiology of the viral hepatitis in Brazil: A scoping review
Source: PLoS One. 2026 Jul 17;21(7):e0353840. doi: 10.1371/journal.pone.0353840 (PMC13379016; doi:10.1371/journal.pone.0353840)
Supplement: S3 Table — (PDF) [file pone.0353840.s003.pdf]

Codebook – Database: Hepatitis scoping review

Variable	Description	Variable type
id	Internal study identifier in the spreadsheet	categorical
title	Title of the study (original language)	categorical
Year_publ	Year of study publication	integer
DOI	Study DOI	categorical
data collection period	Study data collection period (years)	categorical
publication type	Type of publication	categorical
study_design	Study design	categorical
study_coverage	Study coverage	categorical
region	Geographic region of the study (Brazil)	categorical
Sample size	Sample size	integer
Age group	Age group	categorical
gender	Sex/gender information	categorical
race	Race/ethnicity information	categorical
education	Education/schooling information	categorical
hepatitis	Type(s) of hepatitis studied	categorical
Pop_vulnerable	Marker of the target population of the study	categorical
Pop_groups	Population group label	categorical
Anti-HAV total	Serological marker for hepatitis A	continuous numeric variable
Anti-HAV IgM	Serological marker for hepatitis A	continuous numeric variable
Anti-HAV IgG	Serological marker for hepatitis A	continuous numeric variable
another_data_HAV	Other data related to HAV (e.g., genotype)	categorical
HBsAg	Serological marker for hepatitis B	continuous numeric variable
Anti-HBc total	Serological marker for hepatitis B	continuous numeric variable
another_data_HBV	Other data related to HBV (e.g., genotype, anti-HBs)	categorical
Anti-HCV	Serological marker for hepatitis C	continuous numeric variable
another_data_HCV	Other data related to HCV (e.g., genotype)	categorical
Anti-HDV total	Serological marker for hepatitis D	continuous numeric variable
Anti-HDV IgG	Serological marker for hepatitis D	continuous numeric variable
another_data_HDV	Other data related to HDV (e.g., genotype)	categorical
Anti-HEV total	Serological marker for hepatitis E	continuous numeric variable
Anti-HEV IgM	Serological marker for hepatitis E	continuous numeric variable
Anti-HEV IgG	Serological marker for hepatitis E	continuous numeric variable
another_data_HEV	Other data related to HEV (e.g., genotype)	categorical
Id_endnote	Reference ID (EndNote)	categorical
authors	Authors	categorical
